# Production and properties of non-cytotoxic pyomelanin by laccase and comparison to bacterial and synthetic pigments

**DOI:** 10.1038/s41598-021-87328-2

**Published:** 2021-04-20

**Authors:** Faustine Lorquin, Fabio Ziarelli, Agnès Amouric, Carole Di Giorgio, Maxime Robin, Philippe Piccerelle, Jean Lorquin

**Affiliations:** 1 Mediterranean Institute of Oceanology (MIO), Aix-Marseille Université, 163 avenue de Luminy, 13288 Marseille Cedex 9, France; 2 Mediterranean Institute of Marine and Terrestrial Biodiversity and Ecology (IMBE), Aix-Marseille Université, 27 Boulevard Jean Moulin, 13385 Marseille Cedex 5, France; 3 Fédération Sciences Chimiques de Marseille, Aix-Marseille Université, 52 Avenue Escadrille Normandie Niemen, 13397 Marseille, France

**Keywords:** Biotechnology, Biomaterials, Industrial microbiology

## Abstract

Pyomelanin is a polymer of homogentisic acid synthesized by microorganisms. This work aimed to develop a production process and evaluate the quality of the pigment. Three procedures have been elaborated and optimized, (1) an HGA-Mn^2+^ chemical autoxidation (Pyo_CHEM_ yield 0.317 g/g substrate), (2) an induced bacterial culture of *Halomonas titanicae* through the 4-hydroxyphenylacetic acid-1-hydroxylase route (Pyo_BACT_, 0.55 g/L), and (3) a process using a recombinant laccase extract with the highest level produced (Pyo_ENZ_, 1.25 g/g substrate) and all the criteria for a large-scale prototype. The chemical structures had been investigated by ^13^C solid-state NMR (CP-MAS) and FTIR. C_ar_–C_ar_ bindings predominated in the three polymers, C_ar_–O–C_ar_ (ether) linkages being absent, proposing mainly C_3_-C_6_ (α-bindings) and C_4_-C_6_ (β-bindings) configurations. This work highlighted a biological decarboxylation by the laccase or bacterial oxidase(s), leading to the partly formation of gentisyl alcohol and gentisaldehyde that are integral parts of the polymer. By comparison, Pyo_ENZ_ exhibited an M_w_ of 5,400 Da, was hyperthermostable, non-cytotoxic even after irradiation, scavenged ROS induced by keratinocytes, and had a highly DPPH-antioxidant and Fe^3+^-reducing activity. As a representative pigment of living cells and an available standard, Pyo_ENZ_ might also be useful for applications in extreme conditions and skin protection.

## Introduction

**Figure 1:**
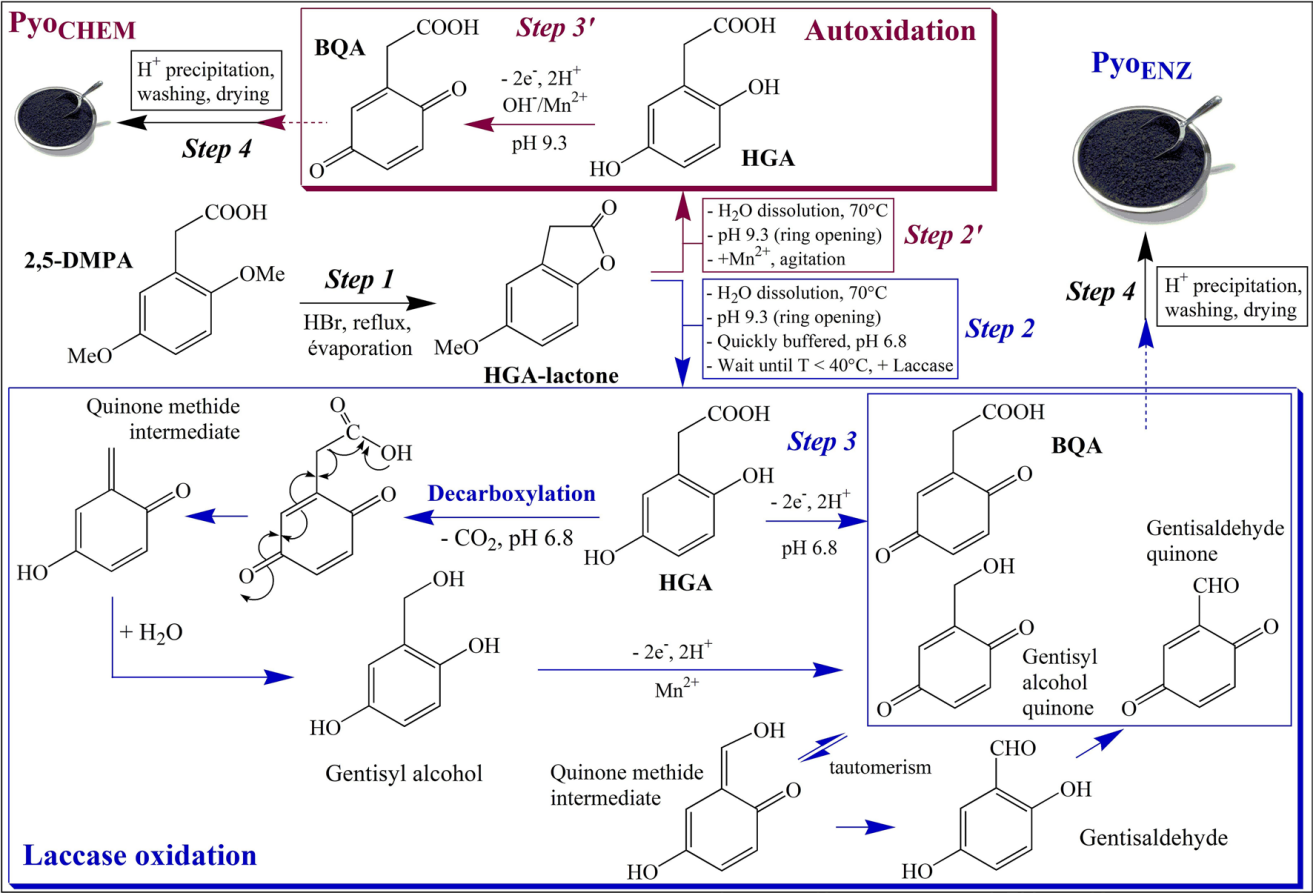
Schematic diagram of the Pyo_ENZ_ process (in blue) that uses a laccase and showing the associated decarboxylation mechanism and the two resulting products identified in the polymer. Comparison to the Pyo_CHEM_ synthesis (abiotic autoxidation, in red). The common precursor 2,5-DMPA could also be synthesized from 2,5-dimethoxyacetophenone by a Willgerodt-Kindler reaction type^25^. Step 4 is the final HCl precipitation followed by washing and drying. Doubling the amount of HBr (step 1) led to incomplete demethylation and the extra formation of 2,5-dihydroxyphenylacetaldehyde (~ 6%) identified from the HPLC-DAD spectrum (λ_max_ 292 nm) and the EI-MS profile (molecular ion [M + 2TMS] at *m/z* 294, characteristic fragments [M – CHO] at *m/z* 265, and [M – CH_2_CHO] at *m/z* 251), similarly to the NIST data bank and previous data^34^. In step 2, the addition of sulfite (Na_2_SO_3_)^25^ was unnecessary because the solution was immediately buffered to 6.8 and the polymerization by the laccase followed (step 3). The alkaline opening of the lactone was essential, indeed the rMt laccase was unable to open the lactone nor demethylate 2,5-DMPA at pH 6.8, even after several days of agitation. BQA, 1,4-benzoquinone acetic acid; gentisaldehyde, 2,5-dihydroxybenzaldehyde; gentisyl alcohol, 2,5-dihydroxybenzyl alcohol; 2,5-DMPA, 2,5-dimethoxyphenylacetic acid; 2,5-DMAPO, 2,5-dimethoxyacetophenone.

Pyomelanin is a natural polymer of homogentisic acid (HGA, 2,5-dihydroxyphenylacetic acid) synthesized through the L-tyrosine pathway by bacteria, fungi, mammals, and plants, and belongs to the heterogeneous group of allomelanins^1^. In living cells, the HGA 1,2-dioxygenase disruption or deletion leads tfigo pyomelanin accumulation^2^ (Fig. 1S). The first property of the pigment is to protect microorganisms from UV light limiting free radicals and ROS generation^3^. The excitation of L-Dopa melanin by UV light produces cell-damaging ROS^4^, whereas the formation of ROS by light has never been reported with pyomelanin. Therefore, pyomelanin increases resistance to light, for instance in *Legionella*^5^. Its antioxidant role has also been demonstrated in *Pseudomonas aeruginosa*^6^, *Burkholderia cenocepacia*^7^, *Aspergillus fumigatus*^2^, and many other strains. Pyomelanin also diminishes the oxidizing stress of the host microorganism, by its high tolerance to H_2_O_2_, as demonstrated in *Ralstonia solanacearum*^8^ and clinical isolates of *Pseudomonas aeruginosa* from infected patients^9^. Moreover, pyomelanin is an antibacterial and antifungal agent against microbial outside attacks, especially pathogenic^10^, reduces biofouling^11^, chelates heavy metals^3,12^, and contributes to microbial pathogenesis as it is associated with virulence in a broad range of pathogenic fungi and bacteria^9,13^. Pyomelanin potentially reduces soluble FeIII in FeII^14–16^, FeII being essential in many bacteria such as *Legionella pneumophila*, that ensure homeostasis by an appropriate Fe^2+^/Fe^3+^ ratio for their survival^17,18^. Consequently, in vivo or in vitro, pyomelanin may serve as a terminal electron acceptor, electron shuttle, or conduit for electrons, a complementary iron acquisition to the siderophore role. Several ways for pyomelanin synthesis may be explored. In microorganisms, two distinct pathways led to HGA formation, through a 4-HPP dioxygenase (4-HPPD; EC 1.13.11.27; route 1), the most described from many bacteria mostly pathogen, and via a 4-HPA-1-hydroxylase (4-HPAH-1; EC 1.14.13.18; route 2) (Fig. 1S). To date, there are very little data on microbial pyomelanin production and yield reminds weak (max. 0.35 g/L^19^), comparatively to the eumelanin pigment produced at 28.8 g/L by a tyrosinase overexpression in a recombinant *Streptomyces kathirae*^20^. The construction of a recombinant 4-HPPD enzyme is not an option because the substrate 4-HPP is expensive. Recently, as opposed to the plant enzyme-based assay, an optimized high-throughput screening assay using human 4-HPPD was constructed using the *E. coli* strain C43 (DE3) supplemented with L-Tyr in the culture medium, a useful tool to find new inhibitors against alkaptonuria disease^21^. However, no pyomelanin yield was reported. On the other hand, the 4-HPAH-1 enzyme responsible for HGA synthesis in *Delftia acidovorans*^22^ and *Azoarcus evansii*^23^, had been partially characterized. In 2008, the sequence of the *D. acidovorans* enzyme had been given by several genomic approaches^24^. The enzyme contains two components, *hpaH* which codes for a flavoprotein NAD(H)-dependent oxidase that transforms 4-HPA in a non-identified metabolite called Z, the second *hpaC* catalyzes the conversion of Z in HGA. *hpaH* and *hpaC* were cloned together, however, no HGA synthesis occurred from these constructions. To date, HGA is very expensive, at about $800/gram. Several methods of chemical synthesis have been developed but the majority not applicable for a large-scale process, the yield being comprised of 50–60% when indicated. Ultimately, the former procedure of DeForrest Abbott and Doyle-Smyth^25^ through HGA-lactone as intermediate, remained the most convenient and proposed in two or three simple steps. For a long time, pyomelanin had been described as the result of the HGA autoxidation catalyzed by Mn^2+^ or Cu^2+^, from neutral to alkaline conditions^26^. For 30 years, several authors hypothesized the action of oxidases, such as the multicopper-dependent laccases (EC 1.10.3.2) generally involved in the polymerization of dihydroxylated phenols^27^. Laccases have also been suggested in that of HGA in *Vibrio cholerae*^28^, *Alcaligenes eutrophus* (now *Cupriavidus necator*)^29^, *Metarhizium anisopliae*^30^, and their role definitively confirmed in *Cryptococcus neoformans*^31^.

At this time, no marketed pyomelanin exists, and a method to furnish acceptable quantities of pigment became necessary. We have therefore developed a large-scale and low-cost production process by reconsidering the chemical synthesis of HGA on the one hand, and the use of laccase on the other. From this optimized enzymatic process, the resulting pigment (Pyo_ENZ_) and its chemical structure and properties had been examined. For comparison, bacterial pyomelanin (Pyo_BACT_) from an induced *Halomonas* culture, the bacterial process, and that issued from the HGA autoxidation (Pyo_CHEM_), the chemical process, was also extensively characterized. Furthermore, the mechanism of HGA polymerization was re-evaluated with a lot of care.

## Results

### Development of an enzymatic process (Pyo_ENZ_)

**Figure 2:**
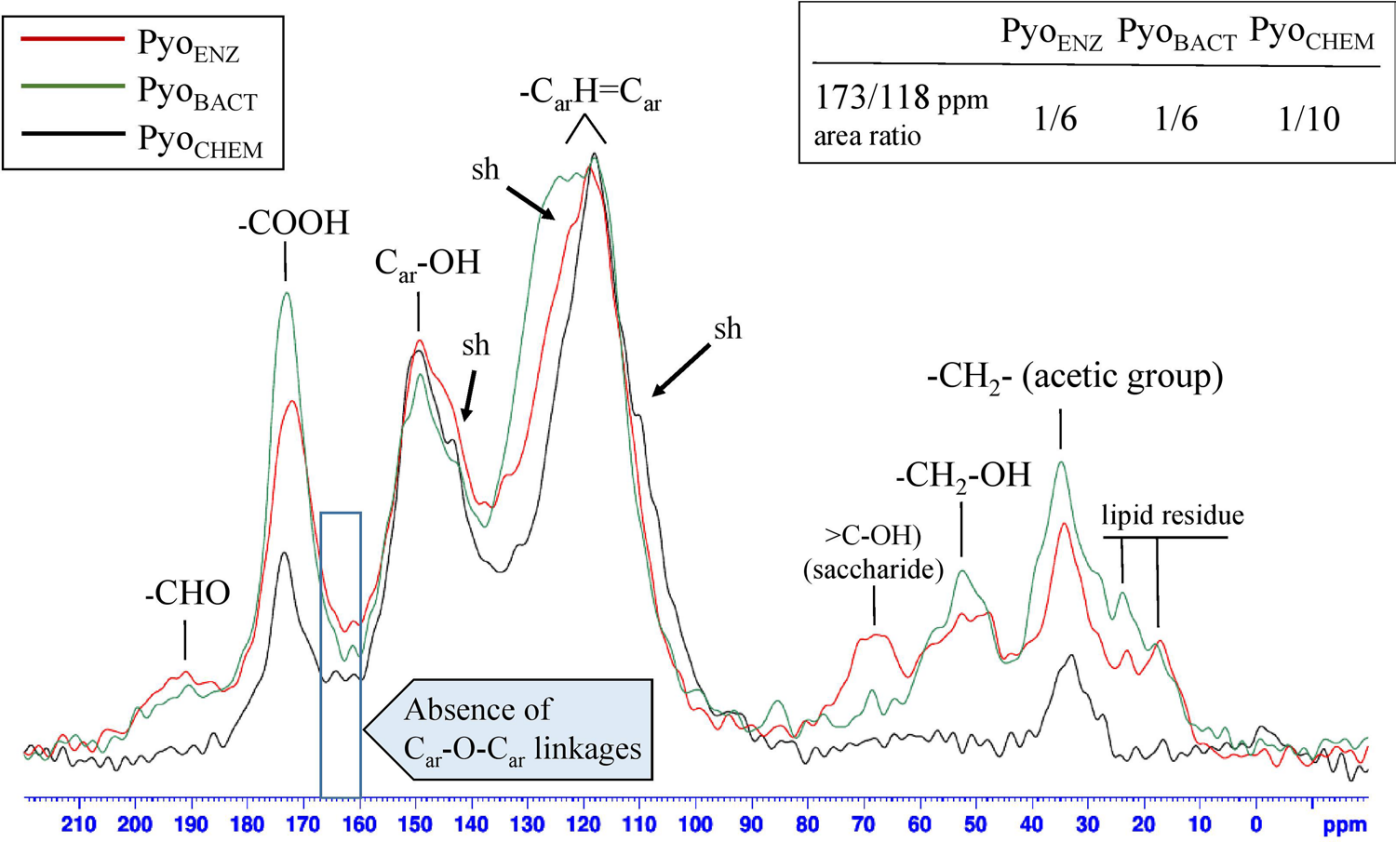
^13^C Cross-Polarization Magic Angle Spinning (CP-MAS) solid-state NMR spectra of the three pyomelanin. The peaks area allowed us to estimate gentisyl alcohol at ~ 9 to 10% and gentisaldehyde at ~ 2 to 3% in Pyo_ENZ_ and Pyo_BACT_. The loss of carboxylic moieties observed on the Pyo_CHEM_ structure (ratio 1/10, black line) was explained by a partial degradation due to the alkaline conditions during the polymerization. Spectra were recorded on an Avance III spectrometer (BRUKER BIOSPIN GmbH, Germany) operated at the Larmor frequencies of 400.43 MHz and 100.70 MHz on ^1^H and ^13^C nuclei, respectively, by using a 4 mm cross-polarization magic angle spinning (CP-MAS) probe head. The zirconia rotors were filled with 80 mg of fine powder polymer or standards. 2048 scans were used to acquire the ^13^C spectra, the acquisition time was 28.7 ms. The ^1^H 90° pulse length and the power level were 3.8 s and 80 kHz, respectively. The spectral width was 35.7 kHz and 2048 points were acquired to describe the free induction decay. A two-pulse phase modulation (TPPM) proton decoupling (75 kHz) was used during the ^13^C acquisition. Spectra were externally referenced to the carbonyl peak of glycine at 176.03 ppm downfield of TMS. No line broadening was applied. All the spectra were acquired using the same receiver gain. sh, shoulder.

**Figure 3:**
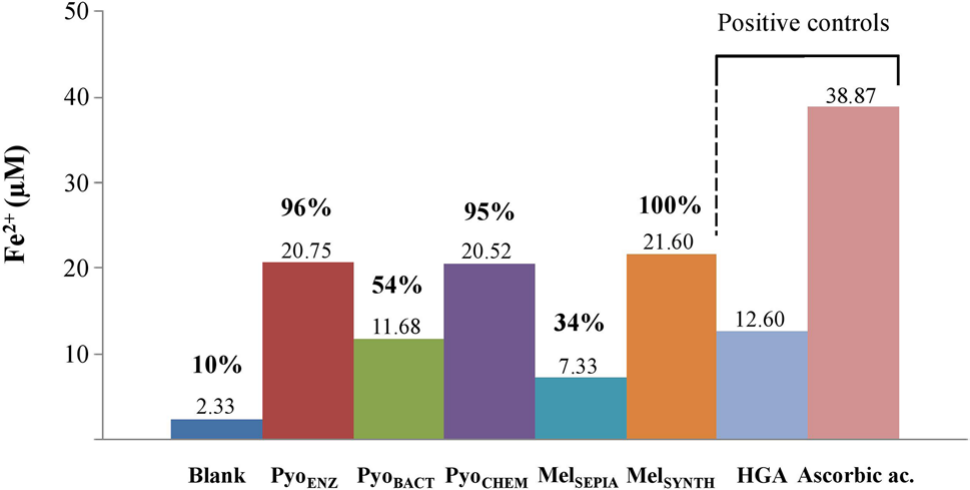
Fe^3+^ reduction by pyomelanin issued from the three processes (Pyo_ENZ_, Pyo_BACT_, Pyo_CHEM_), and comparison to the commercial melanin (Mel_SEPIA_, Mel_SYNTH_). HGA and ascorbic acid were used as positive controls. The Fe^3+^-reducing activity was ada from other methods^17,18,62^. Briefly, 400 μM ferrozine and 120 μM anhydrous FeCl_3_ (final conc.) were extemporaneously mixed in a Tris-HCl 25 mM buffer pH 7.5 (solution A). Then, 5 μL (50 µg) of the melanin stock solutions, each at 10 mg/mL in NaOH 0.05 N, was added in 1 mL of A in closed glass tubes. Blanks were prepared identically without melanin. Mixtures were incubated for 12 h at room temperature and the developed color was measured at 562 nm. As positive controls, 10 µL (44 µg) of ascorbic acid 25 mM solution, and 20 µL (50 µg) of HGA 15 mM, which have defined reduction activity, were also mixed with 1 mL A, incubated, and measured identically. To quantify the activity, a standard curve was generated with known concentrations of ferrous sulfate complexed with ferrozine (solution A), a stock solution of FeSO_4_,7H_2_O 3 mM in milliQ-H_2_O used. After correction, the concentration of Fe^2+^ varied from 0.92 to 13.8 µg, i.e., 6 to 90 µM, and a typical standard equation was: A_562 nm_ = 0.026 x [Fe^2+^ (µM)] + 0.0093. All values were expressed as a mean of three independent experiments, SD < 5% not given to lighten the figure. These experiments suggest that the reducing capacity of HGA is lost after polymerization, and ascorbic acid remains the stronger reducing agent which is however degraded on time.

**Figure 4:**
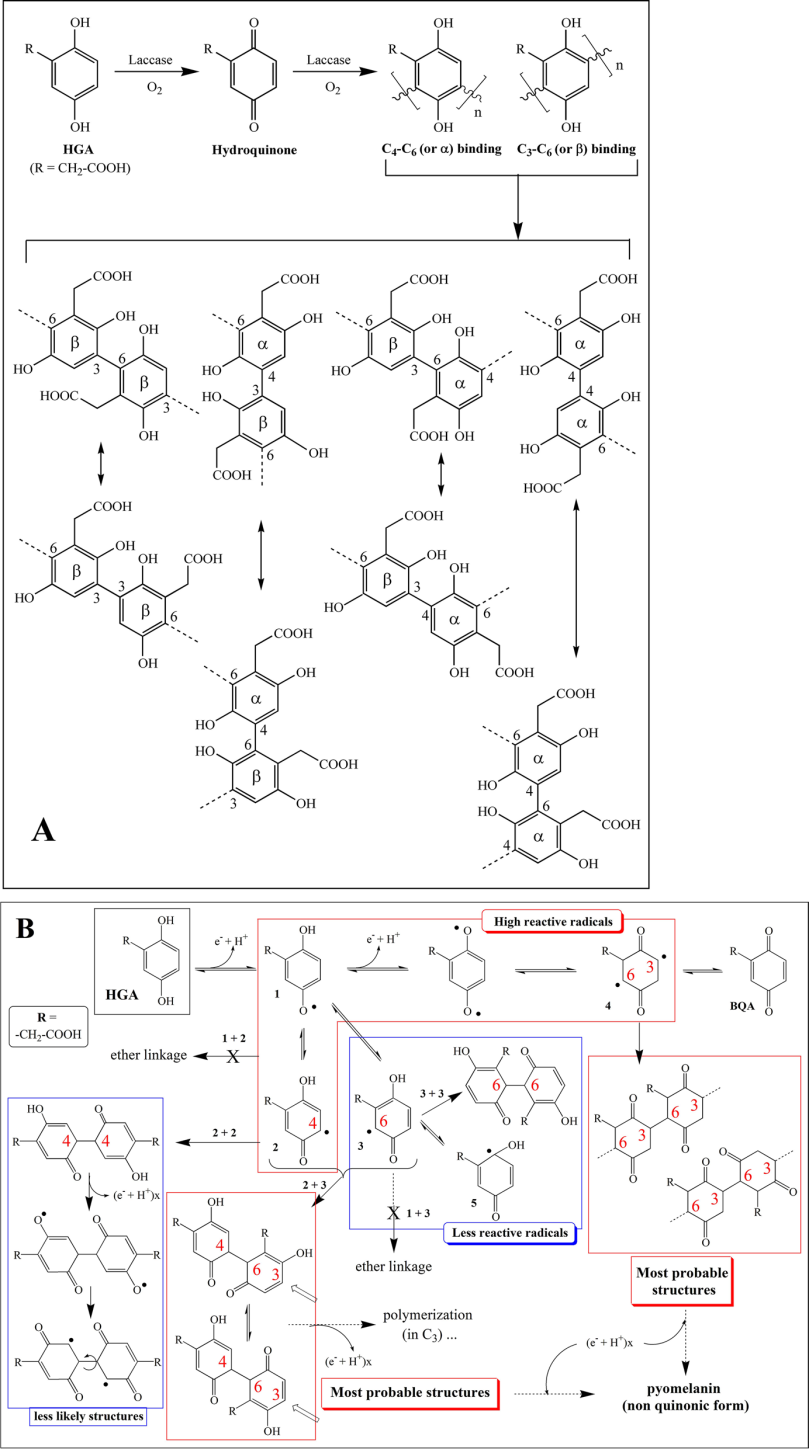
The proposed mechanism of HGA polymerization by the laccase or in abiotic conditions, giving the most probable structure in (**A**) and the most active radical reactions detailed in (**B**). These two figures show the configurations of the C_ar_-C_ar_ links present in the three pyomelanin structures, whereas C_ar_-O-C_ar_ (ether) links are absent as demonstrated by ^13^C solid-state NMR (see text). In these structures, gentisyl alcohol (major) and gentisaldehyde (minor) issued from the decarboxylation mechanism (laccase process, bacteria) are supposed to be incorporated into the polymer in the same manner as HGA radicals at locations of the chain that could not be determined at this time.

To produce high quantities of pyomelanin, appropriate chemical synthesis of HGA has been combined with a polymerization step by a selected laccase in an on-line process (steps 1 to 4, Fig. 1). The first part of the adapted synthesis of DeForrest Abbott and Doyle-Smyth^25^ yielded pure HGA-lactone (step 1, ~ 99% yield) as controlled by HPLC and GC-MS (see spectroscopic data in Methods). The alkaline hydrolysis of the lactone was followed by immediate buffering at pH 6.8 (step 2) and the addition of the rMt laccase extract (step 3) that reacted in two successive stages, the formation of BQA and the polymerization (step 3). In the final step 4, the polymer was precipitated, washed, and dried. The 2,5-DMPA as the starting molecule of the process could be prepared from 2,5-dimethoxyacetophenone^25^, four times less expensive according to various suppliers. Finally, the optimal yield was reached with 40 mM HGA (final conc.) or HGA-lactone equivalent, 17–22 U (~ 23–30 µL) of rMt laccase extract per mL of reaction volume, at pH 6.8 for 24 h agitation (Fig. 2S and 3S). A temperature of 50 °C modified the polymerization rate but did not improve the yield at the end of the incubation time that must be 25 h (Fig. 4S). In these conditions, at 30 °C, the process yielded 1.25 ± 0.11 g of dried Pyo_ENZ_ per g of 2,5-DMPA (mean of 3 experiments).

### Production of pyomelanin (Pyo_BACT_) by an induced wild *Halomonas* strain

A bacterial strain able to compete with the enzymatic process (Pyo_ENZ_) in terms of production yield, was sought. The strategy consisted to select a *Halomonas* species among a large collection, like our previous studies^32–34^. Phenolic compounds in the medium were identified and controlled along with the growth of the induced cultures, the strains preferentially utilized the aromatic over glucose. These halophile bacteria easily grow and have been shown to produce dihydroxy phenols from 4-hydroxyphenylacetic acid (4-HPA), such as HGA in *H. olivaria*^32,34^, *H. venusta*, *H alkaliphila*, and 3,4-dihydroxyphenylacetic acid (3,4-DHPA) in *H. alkaliantartica*, *H. neptunia*, *H. sulfaedris* (this work), and *H*. sp. HTB24^35^, through routes 2b and 3, respectively (Fig. 1S). *H. titanicae* is a γ-proteobacterium isolated on the Titanic wreck, its genome now entirely available^36^, and has been selected for the most intense brown-black color from 5 mM 4-HPA supplemented cultures measured by the A_400 nm_ (this work). The suspected presence of pyomelanin was first confirmed by identification of HGA (λ_max_ 290 nm) only in the exponential phase, but not 3,4-DHPA or other dihydroxyphenylacetic derived compounds, by RP-HPLC-DAD and GC-MS of the TMS-derived metabolites, showing a matched fragmentation spectrum with that of the HGA standard. The strain could not grow in the presence of L-Tyr and was unable to metabolize 2-HPA or 3-HPA, hence suggesting that a 4-HPA-5-hydroxylase or a 4-HPA-6-hydroxylase were not implied, respectively. We concluded that *H. titanicae* was able to produce pyomelanin by direct conversion of 4-HPA to HGA through a 4-HPA-1-hydroxylase (4-HPAH-1, route 2b, Fig. 1S). Following this, pyomelanin production has been optimized. Because 5 mM 4-HPA was rapidly consumed in 2 days and served as an inducer of 4-HPAH enzymes^34,35^, successive additions of well-defined amounts of 4-HPA at different culture times were carried out by following an experimental design procedure (see Methods). Finally, pyomelanin was overproduced in a 500 mL medium by adding 5 mM 4-HPA at starting, then 10 mM after 3 days, in a total culture time of 6 days. In these conditions, *H. titanicae* was able to furnish 0.55 ± 0.09 g Pyo_BACT_ per Liter of culture, a mean of three independent experiments. Relative to the total amount of 4-HPA added, the recalculated yield was 0.241 ± 0.04 g Pyo_BACT_ per g of 4-HPA.

### Production by chemical autoxidation (Pyo_CHEM_)

While pyomelanin issued from the HGA autoxidation has been commonly used^2,28^, the reaction has never been optimized to date. In presence of the transition metal Mn^2+^ to enhance the catalysis^26^, an (HGA)/(Mn^2+^) ratio of 20 for an optimal pyomelanin yield was obtained (see Methods). By applying this ratio, the developed on-line process (Fig. 1, steps 1–2′-3′-4) provided 0.317 ± 0.031 g Pyo_CHEM_ per g of 2,5-DMPA (mean of 5 experiments), a yield four times lower than that of the enzymatic (Pyo_ENZ_) and higher than the bacterial (Pyo_BACT_) process (summarized in Table 2). Despite this, the production of Pyo_CHEM_ remains interesting because the cheapest and easy to implement for use on a laboratory scale.

### Structural data of the three pyomelanin

Better than ^1^H-NMR, solid-state ^13^C-NMR analyses of polymers can provide not only structural features through the resonances of the monomers but also the types of bindings. Pyomelanin issued from the three developed processes, Pyo_ENZ_, Pyo_BACT_, and Pyo_CHEM_ were analyzed by solid-state ^13^C CP-MAS at their optimal signal resolution in conjunction with FTIR experiments.

### Solid-state ^13^C CP-MAS NMR

Spectra were cumulated in Fig. 2, and chemical shifts summarized in Table 1 along with those of pure HGA analyzed in the same conditions. The three spectra exhibited common, typical, and prominent signals in slightly varied positions, at δ 172–173.4 ppm that corresponded to the unprotonated carbon in C-O/C = O of the carboxylic group, then at 149.3–149.4 of the unprotonated carbon (suggested C_5_) of the ring bearing the -OH group, with shoulders at 143.4 for the three pyomelanin (suggested C_2_), and at 118–119 ppm provided by the ethylenic and protonated carbons of the ring (-CH = C-) together in broadband. Less high signals at 33.0–34.8 ppm observed on the three structures represented the saturated aliphatic carbons (-CH_2_-) of the acetic acid moiety. From the three ^13^C solid-state NMR spectra, the main differences are in the region around δ 45–78 ppm, precisely at 52.5 and 67.9 (larger) ppm in Pyo_ENZ_, and 52.5 ppm alone in Pyo_BACT_, whereas these two shifts are absent in Pyo_CHEM_ (Fig. 2). They suggested secondary reactions during the biological BQA polymerization that did not occur during autoxidation of HGA in abiotic and alkaline conditions. By comparison to standard molecules analyzed in parallel, the δ 52.5 ppm shift corresponds to the bi-protonated carbon of the ethanolic moiety (-CH_2_-O-) from 2,5-dihydroxybenzyl alcohol (gentisyl alcohol). Besides gentisyl alcohol, we also noted minor peaks at δ 190.5 and 191 ppm in Pyo_BACT_ and Pyo_ENZ_ respectively, absent in Pyo_CHEM_, and ascribed to an aldehyde group of the end-product 2,5-dihydroxybenzaldehyde (gentisaldehyde) (Table 1). Gentisyl alcohol and gentisaldehyde resulted from a decarboxylation reaction extensively detailed in Fig. 1. With a lot of precautions because solid-state NMR was a semi-quantitative tool, the relative level of the decarboxylation products in the polymers was deduced from areas of the corresponding peaks (Fig. 2), and approximately evaluated at 11–13% (gentisyl alcohol 9–10% + gentisaldehyde 2–3%), these two compounds could not be identified by FTIR. Besides, low signals visualized at δ 17.2–23.1 (Pyo_ENZ_) and 17.4–23.9 ppm (Pyo_BACT_) were attributed to lipid residues provided by the enzyme extract and the culture medium, respectively. The broad signal at δ 67.9 ppm present in Pyo_ENZ_ only had been assigned to the hydroxylated ^13^C of a saccharide moiety (> C-OH) also brought by the laccase extract, whereas it was absent in Pyo_BACT_ probably because the *H. titanicae* medium was not supplemented with glucose. Importantly, the area ratio of the 170/118 ppm resonances for each pyomelanin (Fig. 2, data framed) indicates a correct -CH_2_-COOH substitution for Pyo_ENZ_ and Pyo_BACT_ with a value of 1/6, whereas a loss of the carboxylic moiety on Pyo_CHEM_ structure (ratio 1/10) has occurred. More information on the polymer assembly was necessary to elucidate the mechanism of polymerization and the types of linkage between the rings, *i.e.* C_ar_-C_ar_ (aryl carbon) or/and C_ar_-O-C_ar_ (aryl ether) linkages.

**Table 1:** Summary of the ^13^C CP-MS solid-state NMR chemical shifts and their corresponding assignation for the three pyomelanin pigments, and comparison with standards.

Compound	Protonated carbons			Unprotonated carbons		References
	δ (ppm)	Assignation		δ (ppm)	Assignation	
Pyo_ENZ_	17.2	–CH_3_ (lipid residue)		122 sh	C_ar1_– (suggested)	–
	23.1	–CH_2_–R (lipid residue		143.4 sh	C_ar_–OH	
	34.3	–CH_2_– (acetic group)		149.3		
	52.5 br	–CH_2_–OH (gentisyl alcohol)		172.0	–COOH (carboxylic C)	
	67.9 br	Saccharide moiety (> C–OH)				
	119.0	–C_ar_H=C_ar_				
	191.0 w	–CHO (aldehyde group on C_ar1_)				
Pyo_BACT_	17.4	–CH_3_ (lipid residue)		124 br	C_ar1_– (suggested)	–
	23.9	–CH_2_–R (lipid residue		143.4 sh	C_ar_–OH	
	34.8	–CH_2_– (acetic group)		149.2		
	52.5 br	–CH_2_–OH (gentisyl alcohol)		152 sh	Undetermined	
	118.1	–C_ar_H = C_ar_		173.0	–COOH (carboxylic C)	
	190.5 w	–CHO (aldehyde group on C_ar1_)				
Pyo_CHEM_	33.0	–CH_2_– (acetic group)		143.4 sh	C_ar_–OH	–
	110sh	Undetermined		149.4		
	118.1	–C_ar_H=C_ar_		173.4	–COOH (carboxylic C)	
**Standards**						
HGA	33.9	–CH_2_– (acetic group)		122.3	–C_ar1_–	Data similar to the literature^64^
	116.5	C_ar4_–H	#	146.7	C_ar2_–OH	
	117.0	C_ar3_–H		148.3	C_ar5_–OH	
	120.9	C_ar6_–H		180.9	–COOH (carboxylic C)	
Gentisyl alcohol	55.1	–CH_2_–OH		125.9	C_ar1_–CH_2_OH	Data quite similar to those of Molbase site (www.molbase.com)
	115.9	–C_ar_H=C_ar_(C_ar3,4,6_–H)*		147.1	C_ar2_–OH	
				154.4	C_ar5_–OH	
Gentisaldehyde	119.2	–C_ar_H=C_ar_(C_ar3,4,6_–H)*		125.9	C_ar1_ (–CHO attachment)	Data quite similar to those of Molbase site (www.molbase.com)
	200.6	–CHO (aldehyde group on C_ar1_)		149.0	C_ar5_–OH	
				155.6	C_ar2_–OH	

Car, aromatic carbon; sh, shoulder; br, broad; w, weak.

*Unresolved; # well resolved.

### FTIR analyses

Pyo_BACT_ and Pyo_ENZ_ exhibited very similar FTIR and ^13^C-NMR spectra, hence the study was focused on the Pyo_ENZ_ and Pyo_CHEM_ absorptions (Fig. 5S) noting that the spectrum of Pyo_CHEM_ was better resolved in reason to its less high M_w_ (2,300 Da, Table 2). The peaks at the following wavenumbers and their corresponding structures included the bands for Pyo_ENZ_ and Pyo_CHEM_, respectively at (*i*) 3401 and 3278 cm^−1^ (broad) indicative of the –OH stretch of polymeric structures; (ii) two smaller bands for each compound at 2960 (Pyo_ENZ_) and much more intense at 2925–2927 cm^−1^ (Pyo_CHEM_), which corresponded to stretching vibrations of the aliphatic C_ar_–H groups; (iii) 1711 and 1720 cm^−1^ quite resolved here and ascribed to carbonyl stretching (C=O) of the -COOH group, these bands were however absent on other microbial pyomelanin^29,37^; (iv) 1623 and 1656 cm^−1^ absorptions that were described as typical for aromatic C=C conjugated with C=O groups (quinones), with a stronger response in Pyo_CHEM_; (v) 1384 and 1385 cm^−1^ (both minor) would be assigned to the O–H bond of the hydroxyl groups attached to the ring; and (vi) strong bands at 1197 (Pyo_ENZ_) and 1222 cm^−1^ (Pyo_CHEM_) of the phenolic-OH links.

**Table 2:** Summarized chemical and biological properties of pyomelanin and commercial melanin.

Production yield	Pyo_ENZ_	Pyo_CHEM_	Pyo_BACT_	Mel_SYNTH_	Mel_SEPIA_
	1.25 ± 0.11 g/g 2,5-DMPA	0.31 ± 0.03 g/g 2,5-DMPA	0.55 ± 0.09 g/L 0.24 ± 0.04 g/g 4-HPA	–	–
**Polymer size**					
M_W_ (g/mol)	5400	2300	5700	2000	ND^f^
M_n_ (g/mol)	360	350	470	250	–
M_p_ (g/mol)	3600	2500	4600	1600	–
Ð (M_W_/M_n_)	15.3	6.64	11.9	7.95	–
Monomer composition	HGA, BQA, GA, GALD^a^	HGA, BQA^a^	HGA, BQA, GA, GALD^a^	DHI, DHICA^b^	DHI, DHICA^b^
**Elemental analysis**	(%)	(%)	(%)	(%)	(%)
C	49.15 ± 0.08	54.61 ± 0.11	40.58 ± 0.03	49.69 ± 0.10	34.10 ± 0.03
H	3.40 ± 0.02	2.59 ± 0.10	3.15 ± 0.03	2.83 ± 0.05	3.23 ± 0.02
N	2.75 ± 0.02	0	3.65 ± 0.05	6.34 ± 0.03	6.31 ± 0.08
O^d^	44.70	42.80	52.62	41.14	56.36
C/O	1.10	1.27	0.77	1.20	0.6
C/N	18	–	11	8	5.4
**Formula**^**c**^	C_221_H_140_N_11_O_151_	C_105_H_59_O_61_	C_193_H_179_N_15_O_187_	C_83_H_57_N_9_O_51_	–
**Calc. mass (g/mol)**	5362	2295	5697	1995	–
**Solubility**					
NaOH 0.05 N	≤ 10 mg/mL	≤ 10 mg/mL	≤ 10 mg/mL	≤ 10 mg/mL	≤ 10 mg/mL
DMSO	≤ 0.5 mg/mL	≤ 0.5 mg/mL	≤ 0.5 mg/mL	≤ 0.5 mg/mL	≤ 0.5 mg/mL
Other solvents	Insoluble	Insoluble	Insoluble	Insoluble	Insoluble
**DPPH-antioxidant activity (EC**_**50**_** µg/mL)**^**e**^	27.5	20.23	133.7	25.9	> 300
**ROS scavenging activity (IC**_**50**_** µg/mL)**	82.2 ± 5.5	ND	ND	284.1 ± 12.3	> 500
**UV spectrum range**	200–700 nm	200–700 nm	200–700 nm	200–700 nm	200–700 nm
**Fe**^**3+**^**-reducing activity**					
% Related to Mel_SYNTH_	96	95	54	100	34
In ng Fe^2+^/h/µg^**e**^	5.30	5.24	2.98	5.52	1.94
**Stability to T°C**	≤ 80 °C, 72 h	≤ 80 °C, 72 h	≤ 80 °C, 72 h	ND	ND
**Cytotoxicity (c ≤ 500 µg/mL)**	No	No	No	No	No
**Phototoxicity (PIF)**	No (< 2)	No (< 2)	No (< 2)	No (< 2)	No (< 2)

Since the A_400 nm_ value of solubilized pyomelanin depends on the size of the pigment, a spectrophotometric quantification by surrogate melanin for calibration will not be correct. Weighing precisely the final purified pigment remains the only valuable technique for the quantification of pyomelanin as well as other melanin.

^a^Identified by ^13^C solid-state NMR (Fig. 2 and Table 1).

^b^To confirm the global structure of Mel_SYNTH_ and Mel_SEPIA_, assays on their degradation by alkaline-H_2_O_2_ were conducted similarly to those on human melanin^41^. After centrifugation, 10 μL of the reaction volume was injected into an RP-HPLC(DAD)-QToF system in negative mode and confirmed the L-Dopa melanin structure by the presence of the two markers PTCA and PDCA.

^c^Elemental analysis (C, H, N, S) of the pigments was performed by combustion on a Thermo Finnigan EA 1112 analyzer equipped with an autosampler, all managed by the Eager Xperience software (THERMO SCIENTIFIC, France). The oven was set at 970 °C and the flash combustion at 1800 °C. In the formula C_x_H_y_N_z_, each index was deduced by (x, y, z) = %atom (data from elemental analysis) x M_w_/M_atom_.

^d^The index w (for oxygen O_w_) was deduced from 100% – C – H – N.

^e^Standard deviations were < 5%. Composition (C, H, N) of Mel_SYNTH_ was similar to those described^63^, that of Mel_SEPIA_ close to the reported values^42^ (see sample Com).

^f^Molecular weight of the *S. officinalis* melanin could not be determined on the MCX column eluted in the same conditions (see Fig. 6S). GA, gentisyl alcohol; GALD, gentisaldehyde; ND, not determined.

### Presence of N in Pyo_ENZ_ and Pyo_BACT_

Especially, a reaction of substitution on the C_4_ position of the BQA ring by primary and secondary amines had been reported^38^, such substitutions might occur in biological systems. Here, the volume of the laccase extract added for the Pyo_ENZ_ synthesis seemed insignificant, hence it remained difficult to look for amide or amine bonds from polymers, especially when they are minor. These C-N absorptions were generally encountered at δ 155–180 ppm (amides formed from the carboxylic moiety) and 135–145 ppm (aromatic amines) in ^13^C NMR, mainly at 3000–3500 cm^-1^ (N-H stretching vibrations of aromatic amines) in FTIR, thus drowned in those of the major functional groups. Faced with this inability to detect traces of nitrogenous derivatives by NMR and FTIR, elemental analyses of the three pyomelanin were carried out and showed the presence of N in Pyo_ENZ_ (2.75%) and Pyo_BACT_ (3.65%), as expected none in Pyo_CHEM_, and higher in the indole-based melanin Mel_SYNTH_ (6.34%) and Mel_SEPIA_ (6.31%) (Table 2). The presence of N in Pyo_ENZ_ and Pyo_BACT_ is due to amino acids and amines linked on C_4_ of the HGA rings and provided by the rich laccase extract and the components of the *H. titanicae* culture medium, respectively.

### Linkage determination

Interestingly, the FTIR spectra showed absorption at 1534 cm^-1^ strongly present in Pyo_CHEM_ (Fig. 5S, red) and absent in Pyo_ENZ_ (blue) and Pyo_BACT_. This resonance did not correspond to amides and was rather ascribed to aromatic C_ar_-H. From this remarkable difference, it has been established that Pyo_ENZ_ contains much less C_ar_-H free, which means much more C_ar_-C_ar_ linkages than Pyo_CHEM_. As an important finding from the three pyomelanin ^13^C NMR spectra, C_ar_-O-C_ar_ (aryl ether) linkages were absent (Fig. 2), the related signal generally resonates at around δ 160–167 ppm^39,40^. Hence, the three HGA polymers were assembled by C_ar_-C_ar_ linkages only.

### Alkaline-H_2_O_2_ oxidation assays

This treatment has also been tried on the three pyomelanin and the commercial melanin^41^ (Table 2). While hydrolyzed Mel_SYNTH_ and Mel_SEPIA_ melanin led to the two expected degradation products similarly to the literature^41,42^, pyrrole-2,3-dicarboxylic acid (PDCA, an indicator of DHI-derived units) and pyrrole-2,5,5-tricarboxylic acid (PTCA, of DHICA-derived units), any compound has been detected from the three hydrolyzed pyomelanin by LC(DAD)-MS analyses (see Table 2). Thus, pyomelanin could not be hydrolyzed by such peroxide treatment, even after doubling or lowering the peroxide concentration. A pyrolysis-GC-MS coupling method had been developed to analyze pyomelanin from *Penicillium chrysogenum*^43^ but reported too much heterogeneity to obtain uniform results between samples**.** This method utilizes heat to break the polymer into smaller fragments, such as 4-methoxybenzene acetic acid, 4-methoxybenzene propanoic acid, and other minor phenolic compounds, but not HGA. Unfortunately, this technique failed in Pyo_ENZ_, Pyo_BACT_, and Pyo_CHEM_ with any identifiable compound.

### Physicochemical properties (summarized in Table 2)

All pigments (3 pyomelanin, 2 commercial L-Dopa melanin Mel_SYNTH_ and Mel_SEPIA_) are insoluble in neutral or acidic water as well as many usual organic solvents, entirely soluble in alkaline media such as NaOH (0.05 N minimal conc.). Exceptionally, all the pigments are soluble in DMSO at a concentration that does not exceed 0.5 mg/mL after 24 h agitation. Solubility in H_2_O was not improved after 2 days at 80 °C. As solid form and/or solubilized in alkaline solutions, Pyo_ENZ_, Pyo_BACT_, and Pyo_CHEM_ were stable until 80 °C for 3 days (max. tested) with no degradation products detected by RP-HPLC, and GC–MS analyses, near size modification by GPC/SEC. Molecular weights (M_w_) of the three HGA-pigments were successfully determined and have been found at 5,400 Da (dispersity Ɖ 15.3) for Pyo_ENZ_, 5,700 Da (Ɖ 11.9) for Pyo_BACT_, and a less high M_w_ at 2,300 Da (Ɖ 6.64) and 2,000 Da for Pyo_CHEM_ and Mel_SYNTH_ (Fig. 6S, Table 2), explaining why Pyo_CHEM_ and Mel_SYNTH_ were more rapidly solubilized in DMSO than the others. These M_w_ data were very close to those resulting from the elemental analyses (Table 2), indicating that these pigments were sufficiently purified by successive water and ethanol-washings.

### Antiradical properties

The scavenging ROS activity was studied for Pyo_ENZ_, comparatively to the standards Mel_SEPIA_ and Mel_SYNTH_. UVA induces damage by directly transferring energy or indirectly through ROS generated as primary and secondary radiolytic products^44^. Therefore, the protection by melanin pigment against UVA may be due to their ability to scavenging ROS in the cells. To prove this, a fluorescein-derived compound (DCFH-DA) was used to detect the generation and change of ROS in UVA-visible irradiated keratinocyte cells. Indeed, keratinocytes are a source of ROS that may affect neighboring skin cells, such as melanocytes, and influence the process of melanogenesis or contribute to the progression of vitiliginous lesions. Fluorescence measurements showed that Pyo_ENZ_ effectively scavenged ROS generated by UVA-visible light in the test system with an IC_50_ of 82.2 ± 5.6 µg/mL, while IC_50_ of Mel_SYNTH_ (284.1 ± 12.3 µg/mL) was higher and that of Mel_SEPIA_ very far (Table 3). Thus, the amount of ROS in the cells decreased as the concentration of Pyo_ENZ_ increased, much more efficiently than the concurrent pigment Mel_SYNTH_.

**Table 3:** Effect of Pyo_ENZ_ on scavenging ROS generated by UVA irradiation on keratinocyte cells.

	Concentration (µg/mL)	Fluorescence intensity	ROS release (%)	IC_50_ (µg/mL)
**Pyo**_**ENZ**_	50	331.6 ± 3.3	65.92 ± 2.64	82.2 ± 5.6
	100	244.3 ± 7.7	35.05 ± 1.71	
	250	187.0 ± 6.6	14.86 ± 2.16	
	500	164.0 ± 4.0	6.70 ± 2.28	
**Mel**_**SYNTH**_	50	412.6 ± 2.8	94.51 ± 2.61	284.1 ± 12.3
	100	373.3 ± 11.0	80.67 ± 4.90	
	250	326.0 ± 12.2	63.97 ± 5.13	
	500	231.6 ± 16.4	30.70 ± 6.67	
**Mel**_**SEPIA**_	50	414.6 ± 5.3	95.17 ± 1.70	> 500
	100	393.3 ± 4.9	87.70 ± 3.57	
	250	334.6 ± 19.9	66.92 ± 5.87	
	500	301.3 ± 5.3	55.19 ± 2.86	
Non-irradiated control		145.0 ± 2.4	0	–
Irradiated control		428.3 ± 5.7	100	–

The ROS release was detected by fluorometric measurement and inhibition of the DCFH-DA reagent. Pyo_ENZ_ antiradical activity was compared to that of the melanin standards. Determinations resulted from triplicate assays and IC_50_ values were calculated by the Phototox v2.0 software (ZEBET, Germany). Experimentally, keratinocyte cells (see phototoxicity protocol for preparation) were UVA-irradiated in presence of grads concentration (50–500 μg/mL) of Pyo_ENZ_ and standard melanin (Mel_SYNTH_, Mel_SEPIA_), or not (control). Cells were decanted, washed twice by a 25 mM PBS buffer at pH 7.4, then loaded with 2′,7′-dichlorofluorescein-diacetate (DCFH-DA) to a final concentration of 20 μM, and incubated in dark at 37 °C for 30 min. ROS were then measured by fluorescence intensity of dichlorofluorescein (DCF) at an excitation wavelength of 499 nm and an emission wavelength of 521 nm, in an Infinite M200 Pro fluorescence reader (TECAN, Swiss) equipped with a 1-cm quartz cell. Irradiation was carried out with the Suntest CPS + solar simulator (xenon arc lamp 1100 W, with filters to restrict light transmission below 290 nm and near IR), at a dose of UVA-visible light of 138 kJ/m^2^, the irradiance was at 765 W/m^2^ and irradiation time 3 min. The temperature of the samples was maintained at 4 °C using a water-cooling system linked to the irradiation chamber. Inhibition of DCFH-DA fluorescence (Fluo) was expressed as a percentage as compared to the irradiated and non-irradiated control (without pyomelanin or melanin):

ROS release (%) = 100 × (Fluo_test_ − Fluo_-irr control_)/(Fluo_+irr control_ − Fluo_-irr control_). IC_50_ (μg/mL) values were calculated by the Phototox v2.0 software.

The DPPH-antioxidant activity was rarely reported due to the insolubility of the pyomelanin in organic solvents, and because the stable DPPH reagent reacts at slightly alkaline pH values. The assays were carried out on the three HGA-pigments, along with the two standards (Mel_SEPIA_, Mel_SYNTH_) and common antiradical agents such as Trolox, ascorbic acid, and propyl gallate, all prepared in DMSO. Figure 7S-A and 7S-B indicated that Pyo_ENZ_ (EC_50_ 27.5 µg/mL) and Mel_SYNTH_ (EC_50_ 25.9 µg/mL) have an antioxidant activity equivalent to that of ascorbic acid (29 µg/mL), as already reported for pyomelanin isolated from *Pseudomonas stutzeri* strain BTCZ10 and *Pseudoalteromonas lipolytica* BTCZ28^45,46^. The degradation of pyomelanin by enzymes or microorganisms has never been described to date, whereas ascorbic acid is rapidly metabolized and was thought to act as a pro-oxidant when the glutathione pool is depleted^47^, a feature that must also be controlled in the case of pyomelanin. Barely better than Pyo_ENZ_, Pyo_CHEM_ EC_50_ was 20.0 µg/mL, while EC_50_ Pyo_BACT_ was found much higher at 130.0 µg/mL (Fig. 7S-A). Whatever, Pyo_ENZ_, Pyo_CHEM_, and Pyo_BACT_ exhibited much higher DPPH-antioxidant activities than pyomelanin isolated from the *Yarrowia lipolytica* strain W29 (EC_50_ 230 μg/mL^48^), and far from eumelanin from *Sepia officinalis* (Mel_SEPIA_, > 300 µg/mL, this work) and the synthetic butylated hydroxytoluene (BHT, EC_50_ 722 μg/mL^49^). Although the trihydroxylated benzoic ester, propyl gallate (EC_50_ 4.2 µg/mL) (Fig. 7S-B), was one of the leading dietary antioxidants, it induced DNA damages^50^; BHT was also found cytotoxic^51^, hence their use notably in the food industry became restricted.

### Electron-transfer efficacy

By an adapted ferrozine assay, Pyo_ENZ_, Pyo_CHEM_, and Mel_SYNTH_ exhibited equivalent and highest Fe^3+^-reducing activity among the five polymers tested (Fig. 3). From these data, the equivalent Fe^3+^-reducing activity of Pyo_ENZ_ and Pyo_CHEM_ could not be explained, while Pyo_ENZ_ and Pyo_CHEM_ have a different M_w_ (Table 2), thus none the same number of -OH and carboxylic groups, and even if gentisyl alcohol and gentisaldehyde (at ~ 11 to 13%) are present in Pyo_ENZ_ structure only. Comparatively to Mel_SYNTH_ (100%), the reducing activity in decreasing order was Pyo_ENZ_ (96), Pyo_CHEM_ (95), and to a less extent Pyo_BACT_ (54) and Mel_SEPIA_ (34). Because Pyo_ENZ_ has the best production yield and is dedicated to potent applications, its Fe^3+^-reducing activity was evaluated at 1.73 µM per hour related to 50 µg of pigment, *i.e*. 5.30 ng Fe^2+^/h/µg.

### Cytotoxicity

For applications with pyomelanin as an ingredient for cosmetics or pharmaceutical preparations, cytotoxicity toward human keratinocytes has been evaluated by the vital dye NR penetration technique, from pigment prepared in alkaline solutions at dilutions which in no way modified the pH of the assay. Keratinocytes are the most abundant cells of the epithelial layer of the skin and are used as a part of the 3D skin model for the assessment of the toxic hazard of cosmetic ingredients. No reduction of the metabolic activity of the cells was observed as compared to the non-treated cells, thus formally postulating the absence of toxic effect on skin cell metabolic activity for Pyo_ENZ_, Pyo_BACT_, Pyo_CHEM_, Mel_SYNTH_, and Mel_SEPIA_, until 500 µg/mL (Table 2). Furthermore, using the normalized OECD protocol commonly used for cosmetology product evaluation, the three pyomelanin and the two standard melanins were found non-phototoxic (PIF < 2) (Table 2).

## Discussion

### The laccase process is the most efficient provider of pyomelanin

Comparison of three realistic strategies by optimized production of HGA autoxidation (Pyo_CHEM_), induced bacterial culture (Pyo_BACT_), and for the first time using a recombinant laccase (Pyo_ENZ_) was undertaken. Pyo_ENZ_ has been obtained at the highest level, 1.25 g per g 2,5-DMPA, a yield > 1 g/g due to compounds linked and brought by the concentrated enzyme extract. This procedure meets all the criteria to design a large-scale prototype, high-efficient, cheapest, with mild conditions, and without sterility constraints that are essential in the case of microbial cultures. HGA-lactone was easily prepared from 2,5-DMPA, or even from 2,5-dimethoxyacetophenone to reduce the costs by an additional reaction of Willgerodt-Kindler^25^. Despite the great number of extensive works on pyomelanin-producing microorganisms, to date there have been only three reported quantifications of the pigment, first with the wild yeast *Yarrowia lipolytica* that furnished 0.035 g/L of culture^52^, second 0.173 g/L culture of the *Shewanella algae* BrY strain supplemented by 2 g of L-Tyr/L^14^, and third 0.35 g/L by random mutagenesis of *Pseudomonas putida*^19^. In this work, an induced culture of *H. titanicae* was shown to convert 4-HPA to HGA by a 4-HPA-1-hydroxylase (4-HPAH-1) at the best microbial yield to date, 0.55 g/L culture, a feature confirmed by the presence in its genome of the related *hpaH/C* genes (unpublished). Such bioconversion generally occurred with less energy consumption, and for these reasons more efficiently. It seems reasonable to assume that the bacterial (Pyo_BACT_) and the chemical (Pyo_CHEM_) processes will never be able to compete with the laccase process (Pyo_ENZ_) in terms of production, except maybe by developing a recombinant overproducing microorganism. From these results, the ability of *H. titanicae* to synthesize pyomelanin from 4-HPA and the property of the pigment to reduce Fe^3+^, raise the question of the survival of the bacterium at 4,000 m depth by maintaining a Fe^3+^/Fe^2+^ ratio.

A few remarks are worth noting about the oxidation of HGA. Besides the biological implications of metal-catalyzed oxidations, true autoxidation of biomolecules does not occur in biological systems, instead, this autoxidation is the result of transition metals bound to these biomolecules^53^. By analyzing the Pyo_CHEM_ structure, surprisingly the ^13^C solid-state NMR spectrum revealed an unexplained loss (~ 40%) of carboxylic moiety during the alkaline Mn^2+^-autoxidation of HGA without observable by-products of this degradation and hence contributes to the low pyomelanin yield. To date, the in vitro polymerization of HGA by a laccase has never been studied before. Here, the rMt laccase had been found to efficiently catalyze the HGA polymerization in terms of yield, and still confirmed the involvement of these oxidases in biological environments. It should be noted that pyomelanin-forming bacteria generally grow at pH 6–7, while the autoxidation is optimal at pH 8–9, one more element in favor of the laccase(s) action in living cells. The rMt enzyme supplied as a rich and concentrated extract is largely available and one of the cheapest in the market. Partial purification by ultrafiltration of the rMt extract would be an additional stage unnecessary. Indeed Aljawish et al.^54^ showed that, if the brown color decreases after UF (⁓90%), it eliminates only 2.5-fold of total proteins and the specific activity of the UF-enzyme increased by only 2.1-fold. Other laccases had also been assayed in parallel. At their optimal parameters, we evaluated that the *Trametes versicolor* enzyme furnished ~ twofold less pyomelanin than rMt, while the purified recombinant *Pycnoporus cinnabarinus* laccase gave a quite similar yield, *i.e.* 1.1–1.2 g pyomelanin per g of substrate, at pH 5 in an acetate buffer.

The molecular weight of pyomelanin rarely reported was first evaluated by GPC/SEC at 3,000 Da for the pigment of the bacterium *Alcaligenes eutrophus*, and at 1,700 Da for autoxidized HGA, however, using unconventional PEG/PEO standards^29^. Turick et al*.*^14^ estimated the size of *Shewanella algae* BrY pyomelanin ranging from 12 to 14 kDa, however by high-speed sedimentation and with proteins for calibration. In this work and as a suitable method in an alkaline eluent, the optimized processes led to close M_w_ of 5,400 and 5,700 Da for Pyo_ENZ_ and Pyo_BACT_, respectively (Table 2), a size much higher than those of laccase-synthesized polymers of catechol (M_w_ 1,268 Da), resorcinol (1,489 Da), and hydroquinone (1,157 Da)^27^.

### Biological pyomelanin is a C_ar_-C_ar_ assembly polymer that contains two decarboxylation-issued products

Because alkaline-H_2_O_2_ hydrolyses and pyrolysis experiments failed, the chemical structure of the three pyomelanin was determined by ^13^C solid-state NMR, and partly confirmed by FTIR analyses. Like the hydroquinone polymerization^27^, C_ar_-C_ar_ bindings between the rings predominated in Pyo_ENZ_, Pyo_BACT,_ and Pyo_CHEM_, a finding deduced from the absence of C_ar_-O-C_ar_ (ether linkages) resonance in the NMR spectra of these polymers. The reactions that govern the polymerization of HGA by the rMt laccase were proposed in Fig. 4A and showed two main suggested assembly modes, C_4_-C_6_ (α-bindings) and C_3_-C_6_ (β-bindings), giving preference to the C_3_-C_6_ mode because of less subject to steric effects. Based on the NMR data, it was not possible to differentiate between the eight possibilities (Fig. 4A). The mechanisms of polymerization through radical reactions have also been proposed in Fig. 4B, considering the high reactivity of the primary phenoxy radicals in favor of aryl radicals and still showing C_3_-C_6_ and C_4_-C_6_ linkages as the most probable structures for pyomelanin. In any case, analytical techniques are still not able to deliver the exact structure and the location of the minor HGA derivatives in the polymer, this is the most problematic for all melanin and especially pyomelanin. Nonetheless, there is still to understand the mechanism of polymerization of HGA, particularly the relationships with the laccase structure. In this work, we notably reported a biological decarboxylation from BQA and caused by the action of the rMt laccase or suggested bacterial oxidase(s) (Fig. 1). Such a mechanism led to the formation of gentisyl alcohol and gentisaldehyde representing about 11–13% of the total components, and which polymerized together with BQA. Until today, HGA decarboxylation was attributed to an abiotic reaction from BQA at acidic pH (near 4–5), forming gentisaldehyde as the major product along with minor gentisyl alcohol^34^. In this work, Pyo_BACT_ and Pyo_ENZ_ contained both products, but in reverse order of level, gentisyl alcohol (major) and gentisaldehyde (weak compound). No decarboxylation was observed during the abiotic and alkaline synthesis of Pyo_CHEM_.

### The pyomelanin Pyo_ENZ_ for multiple applications

In addition to a DPPH-antioxidant activity equivalent to ascorbic acid, a high thermostability over time, a non-degradability in cells, Pyo_ENZ_ efficiently scavenges ROS from irradiated human keratinocytes much better than the concurrent Mel_SYNTH_ (Table 3). Comparatively and with a similar technique, 400 µg/mL of L-Dopa melanin isolated from *Pseudomonas maltophilia* has been reported to almost scavenge ROS totally from UVA-induced fibroblast cells^55^. Human eumelanin and pheomelanin photogenerate ROS meanwhile they photoconsume O_2_ and are protective against skin cancer^56^. Nevertheless, they photochemically generate melanin degradation products that are responsible for sunlight-induced melanoma formation by inducing cyclobutane-pyridine dimers (CPDs) from DNA^57^. In contrast, strong irradiation of Pyo_ENZ_ in solution did not generate degradation products, UV-visible spectroscopy, GPC/SEC, and RP-HPLC being reliable techniques to determine that Pyo_ENZ_ is also a photostable polymer. At first glance, the two decarboxylation products in Pyo_ENZ_ and Pyo_BACT_ structure did not seem to influence the Fe^3+^-reducing activity (Fig. 3). In any case, ferric-reducing activity may be considered as a marker of the redox cycling nature of pyomelanin, a property that might be used to conduct electricity like an electronic-ionic hybrid conductor. It was advanced that only a few femtograms per cell were assumed to be enough amount for electron-transfer in bacterial systems^3,14,16^. Consequently, Pyo_ENZ_ could be exploited as a hyperthermostable and Fe^3+^-reducing agent, and for bioelectronic applications better than melanin^58^. As an evident cosmetic ingredient and consistently with recent reports on microbial pyomelanin from *Yarrowia lipolytica*^48^ and *Pseudoalteromonas lipolytica* BTCZ28^46^, all tested at a 100 µg/mL, Pyo_ENZ_ was found non-cytotoxic and non-phototoxic on keratinocyte cells, until 500 µg/mL.

## Conclusions

Pyomelanin issued from the three processes has different properties, giving a large priority to Pyo_ENZ_ that can now be produced in interesting yield and at low cost. The pigment efficiently scavenges ROS, exhibits high DPPH-antioxidant activity, is non-degradable, photostable, non-toxic, and can be stocked indefinitely without any precaution. As a representative pigment of microbial pyomelanin, Pyo_ENZ_ becomes an available standard for laboratories, might be used for applications that require extreme conditions, as an electron-transfer agent, why not for energy storage, and exploited for skin protection, assuming it cannot penetrate the blood skin vessels.

## Methods

### Chemicals and enzymes

Solvents of mass spectrometry grade were supplied by BIOSOLVE (Dieuze, France), media for human keratinocytes, and mouse fibroblasts cultures from DUTSCHER (Brumath, France), main chemicals including standard melanin from SIGMA. Natural melanin (Mel_SEPIA,_ reference M2649) consists of purified eumelanin from the ink of *Sepia officinalis*. Synthetic melanin (Mel_SYNTH_, M8631) is an L-Dopa melanin obtained from L-Tyr in presence of H_2_O_2_. HGA and HGA-lactone were used as standards for HPLC and the GC-mass data bank. The *Aspergillus* sp. laccase (SAE0050) consisted of a highly concentrated and brown miscible-water solution (density 1.15 g/mL, stored at 4 °C) obtained by submerged fermentation of the recombinant *Myceliophtora thermophile* laccase expressed in *Aspergillus oryzae*. This enzyme originally furnished by Novozym under reference 51,003, was re-named here ‘laccase rMt’. Purified laccases from *Pycnoporus cinnabarinus* (recombinant, a gift of The Fungal Biodiversity and Biotechnology Laboratory, Marseille^59^) and *Trametes versicolor* (SIGMA, 38429) were also used.

### *Halomonas* strain selected and growth conditions

The strain *Halomonas titanicae* was provided from the DSMZ collection (Germany), isolated and taxonomically characterized in 2010^43^, and compared in our laboratory among a large collection of *Halomonas* spp.^32^ The strain was grown in a shaker (130 rpm) at 30 °C in a basal medium containing (in g/L), yeast extract 1.0, NaCl 20, KH_2_PO_4_, K_2_HPO_4_ 0.6, NH_4_Cl 1.0, MgCl_2_·6 H_2_O 10, CaCl_2_·2 H_2_O 0.1. The pH was adjusted to 7.0 with a 4 N NaOH solution. Aliquots (25 and 500 mL) were dispensed into flasks and sterilized by autoclaving at 120 °C for 20 min. Ca- and Mg-chloride stock solutions were sterilized separately, and the accurate volumes were added to the medium. L-Tyr and 4-hydroxyphenylacetic acid (4-HPA) stock solutions (250 mM in milliQ-H_2_O, neutralized, heated moderately, sterilized by 0.2 µm pore size filtration) were added before inoculation at 5 mM final concentration. The strain was pre-cultivated twice for 2 days each, in 25 mL of the same saline basal medium containing either L-Tyr or 4-HPA. The preculture served as inoculum at 10% (v/v) for the culture in 500 mL volume that was agitated at 150 rpm, until the A_400 nm_ no longer changed. To overproduce pyomelanin, 500 mL cultures supplemented by repeated addition of 4-HPA amounts were carried out using the experimental design AZURAD software (a company of Marseille). The 8 experiments resulted from the defined parameters, the response Yi (mass of pyomelanin per Liter of culture), and the 6 entry parameters, X_1_ for the 4-HPA concentrations added (2, 5, and 10 mM), and X_2_ for the time of supplementation (at 0, 2 or 3 days of growth). An experimental domain of cubic form was chosen and a second-degree polynomial model applied^60^.

### Process for the production of pyomelanin (Pyo_ENZ_) by the rMt laccase

The first part of the procedure consisted of an adapted HGA synthesis^25^. The second part is the polymerization step by the rMt laccase. The starting compound 2,5-dimethoxyphenylacetic acid (2,5-DMPA) 5 g was solubilized in 40 mL of 48% HBr and refluxed gently for 4.5 h in a 100 mL-bicol flask provided with a refrigerant maintained at 10 °C. The resulting deeply red solution was evaporated to dryness *in vacuo*, the residue (3.80 g, 99.7% yield, 99.8% purity) identified as HGA-lactone following its UV spectrum (λ_max_ 232, 289 nm, bands slightly lower than that of HGA), elution in RP-HPLC (retention time 4.2 min), and GC–MS analyses of the TMS-derived compound (rt 16.3 min), similarly to the standard. In the second step of the procedure and typically, 1.0 g of HGA-lactone was dissolved by agitation in 130 mL hot milliQ-H_2_O (70 °C), stayed 3–5 min and few drops of NaOH 2 N added until pH 9.3 (pH-meter) to hydrolyze the lactone into HGA (in HPLC-DAD, rt 2.7 min, λ_max_ 290 nm), complete ring-opening was ensured by analysis of a 5 µL sampling diluted 10 × in MeOH. Immediately after, 35 mL of Na-phosphate buffer 0.3 M pH 6.8 were added, the concentration of HGA and buffer at this stage was 40 mM and 65 mM, respectively. Once the temperature of the solution has reached 30–40 °C, 3–4 mL of concentrated laccase rMt were added (2250–3000 U in total), the enzyme activity was 750 U/mL (SD < 5%) as determined by the syringaldazine assay (see Fig. 2S, Additional information). Then the mixture was agitated at 130 rpm in dark at 30 °C for 48 h. The formed brown-black pigment was further precipitated by adding 34 mL of HCl 37% (2 N final concentration), agitated for 2 min, and stayed for 24 h, at ambient temperature in dark. The precipitated pyomelanin was centrifuged, washed with milliQ-H_2_O and ethanol, dried, and weighed as previously for Pyo_BACT_ and Pyo_CHEM_ (Fig. 1, step 4). The yield of the process was determined as a mean of three independent preparations from 2,5-DMPA. For optimal pyomelanin yield, laccase activity and HGA concentration to be used were determined in 4 mL glass vials tightly closed and containing 500 µL of Na-phosphate 100 mM buffer pH 6.8 (final 50 mM), 50 to 500 µL (5–50 mM) of HGA 100 mM stock solution in milliQ-H_2_O, 5–40 µL laccase rMt extract (3.75–30 U), and milliQ-H_2_O (qsp), in one mL total volume. Assays were incubated at 30 °C or 50 °C, at incubation time varying from 1 to 30 h, under 120 rpm agitation in dark. The enzyme activity was stopped by placing the tubes in a boiling water bath for 10 min, and pyomelanin content was evaluated by spectrophotometry (see below pyomelanin monitoring). Each point value resulted from triplicate tubes and a mean ± SD.

### Bacterial (Pyo_BACT_) and chemical (Pyo_CHEM_) pyomelanin preparation

Bacterial cultures at the stationary phase were centrifuged (8,500 g, 30 min), pyomelanin (Pyo_BACT_) in the supernatant was precipitated by the addition of 2 N HCl (final conc.), and the solution left to rest for 24 h, at room temperature in the dark. After centrifugation (8,500 g, 20 min), the brown-black pellet was washed successively with milliQ-H_2_O (3×) and ethanol (1×), centrifuged, dried at 70 °C for 2 days, weighed, and powdered as fine particles before storage in glass vials at room temperature. Chemical pyomelanin (Pyo_CHEM_) was prepared from 2,5-DMPA (1 g, 5.1 mmol) that was demethylated by HBr (reflux) and giving HGA-lactone by rotative evaporation. The lactone was then dissolved in hot H_2_O identically to Pyo_ENZ_ preparation (see previously), and the aqueous HGA (0.84 g, 98.1% yield, evaluated by HPLC with pure HGA as calibrant) formed by alkaline treatment at pH 9.3. Autoxidation of HGA was then continued in presence of 5 mM MnCl_2_, 4H_2_O, the solution agitated in dark for 3 days with a barrel at 30 °C, the pigment precipitated with 6 N HCl (final conc.), left to sediment for 12 h, and centrifuged (8,500 g, 20 min). The light-brown colored supernatant indicated the presence of many oxidized compounds that could not precipitate by increasing the acid until 10 N. After washing and drying, the pelleted Pyo_CHEM_ was weighed and stored as for the Pyo_BACT_ and Pyo_ENZ_ pigments. To determine the optimal (HGA)/(Mn^2+^) ratio, concentrations of HGA (1–300 mM) and MnCl_2_ (0.5–20 mM) were assayed in the same manner, the black-brown solutions diluted 50 × in NaOH 0.1 N and absorbance read at 400 nm (A_400 nm_).

### Homogentisic acid and gentisyl alcohol syntheses

Alkaline hydrolysis of 1.0 g of HGA-lactone in hot 130 mL H_2_O was immediately acidified until pH 5 with drops of HCl 37%, followed by the addition of 6.5 mL of a saturated NaCl solution (5% v/v final conc.). Then HGA was extracted 3 × in a funnel with AcOEt, the whole organic phase washed 1 × with milliQ-H_2_O, clarified with solid Na_2_SO_4_, filtered, and evaporated to dryness. To eliminate residual BQA, the dried HGA was dissolved in 0.1% HCOOH and applied on a glass column (20 mL Luer-lock tip syringe mounted with a vacuum flask and a pump) containing 10 cm^3^ of Lichroprep RP_18_ (from SIGMA) previously conditioned with MeOH and acidified H_2_O. After washing by 2 vol. of acidified H_2_O, HGA was eluted by a mixture of MeOH-acidified H_2_O (1:9, v/v), evaporated to dryness, and resulted in a 99.9% purity light grey HGA (yields ~ 70 wt%), as determined by RP-HPLC and GC-MS analyses, indicating that recrystallization was not necessary. As standard for NMR analyses, pure gentisyl alcohol was synthesized from gentisaldehyde by NaBH_4_-reduction in tetrahydrofuran (yield 49%)^61^, purity confirmed by GC-MS and ^1^H-NMR in *d*_6_-DMSO.

### Cytotoxicity

The viability of cells exposed to melanin was expressed as the concentration-dependent reduction of the vital dye Neutral Red (NR) uptake in intracellular lysosomes. Assays were carried out with the three prepared pyomelanin and the two melanin standards (Table 2), all prepared at 10 mg/mL in NaOH 0.05 N (stock solution). Human epidermal keratinocytes neonatal cells were maintained in a complete keratinocyte serum-free medium (Panserin 412, from DUTSCHER) supplemented with bovine pituitary extract (30 µg/mL), recombinant epidermic growth factor (rEGF, 0.2 ng/mL), and an antibiotic cocktail of 10 U/mL penicillin-100 µg/mL streptomycin. Precultures were seeded into 96-well plates (0.2 mL per well) at 1.10^5^ cells/mL concentration. After incubation at 37 °C (5% CΟ_2_) for 24 h until semi-confluent, the medium was decanted, replaced by 200 µL of complete medium containing the melanin (8 concentrations, 0–500 µg/mL), and cells were incubated again for 24 h. After removing the medium, cells were washed, placed into the NR medium (50 µg/mL NR in the complete medium), and incubated for 3 h (37 °C, 5% CO_2_). The medium was removed, cells were washed three times with 0.2 mL of HBSS (Hank's Balanced Salt Solution, from DUTSCHER) to eliminate the excess dye, and 50 µL per well of a distaining solution (50% ethanol, 1% acetic acid, 49% milliQ-H_2_O) was added. The plates were shaken for 15 min at room temperature in the dark. The membrane damage degree, *i.e*. the increase of released NR, was determined by the A_540 nm_ in an Infinite M200 Pro (TECAN, Swiss) reader. The results obtained for wells treated with the pigment were compared to those of untreated (100% viability) and converted to percentage values. Cell viability was calculated as Viability (%) = [A_540_ (test well) — A_540_ (blank)] / [A_540_ (negative control) — A_540_ (blank)]. The concentration of the pigment causing a 50% release of NR as compared to the control culture (IC_50_, in µg/mL) was calculated by non-linear regression analysis using the Phototox v2.0 software (ZEBET, Germany).

### Phototoxicity

The in vitro and normalized 3T3 NRU assay (OECD number 432) was used. Balb/c 3Τ3 mouse fibroblasts (3Τ3-L1, ATCC CL-173, from US type Culture Collection) were grown in DΜΕΜ supplemented with L-glutamine 4 mM and 10% of inactivated calf serum, seeded into two 96-well plates (0.1 mL per well) at 1.10^5^ cells/mL concentration, and incubated (37 °C, 5% CΟ_2_) for 24 h until semi-confluent. The medium was decanted and replaced by 100 µL of HBSS (see before) containing the appropriate pigment concentrations (8 concentrations, 0–500 µg/mL), then cells were incubated (37 °C, 5% CO_2_) in the dark for 60 min. From the two plates prepared for each series of pigment concentrations and the controls, one was selected, generally at random, for the determination of cytotoxicity without irradiation (− Irr), and the other for the determination of phototoxicity with irradiation (+ Irr). For each set of experiments, a negative control (in HBSS) and positive control (chlorpromazine final concentrations from 1 to 100 µg/mL (− Irr) and 0.01 to 1 µg/mL (+ Irr), diluted in ethanol) were performed. The percentages of cell viability were calculated as previously (cytotoxicity). Irradiation was performed with a solar simulator Suntest CPS^+^ (ATLAS MATERIAL TESTING TECHNOLOGY BV, Lochem, Netherlands) device equipped with a xenon arc lamp (1100 W), a glass filter restricting transmission of light below 290 nm, and a near IR-blocking filter. The irradiance was 750 W/m^2^ corresponding to 4.5 J/cm^2^ for one-min irradiation (0.41 J/cm^2^ of UVA and 4.06 J/cm^2^ of visible light). The Photo-Irritation-Factor (PIF) defined by the ration IC_50_ (− Irr) / IC_50_ (+ Irr) was expressed to finalize the results. Based on validation studies (OECD 432 guideline), a test substance exhibiting a PIF < 2 predicts no phototoxicity, 2 < PIF < 5 a probable, and PIF > 5 a phototoxicity.

### Photostability of Pyo_ENZ_ in solution

It was evaluated on 4 mL glass-closed tubes containing 3 mL each of Pyo_ENZ_ solution at 0.05, 0.1, and 0.5 mg/mL NaOH 0.05 N. The tubes were placed horizontally and irradiated in the Suntest CPS^+^ solar simulator, respecting the ICH Q1B guidelines (European Medicines Agency). A strong irradiance by the xenon lamp was maintained with light energy of 550 W/m^2^ during 1 h (*i.e.* 200 J/cm UVA-visible irradiation). Changes in the polymer structure were monitored by UV-visible spectroscopy from 200 to 700 nm and GPC/SEC (see Fig. 6S), comparatively to non-irradiated samples.

### Metabolites identification, pyomelanin monitoring

Phenolic compounds along the three processes were identified by RP-HPLC-DAD and GC-MS according to our previous works^33–35^. To control the pigment formation during the bacterial culture and for optimization of the processes, the black-brown solution was diluted 20 and 50 × in NaOH 0.1 N (qsp 1 mL), respectively, and absorbance (A_400 nm_) read against the same alkaline reference^2^.

## Supplementary Information

Supplementary Information

## Abbreviations

DPPH: 1,1-Diphenyl-2-picrylhydrazyl
FTIR: Fourier Transform InfraRed spectroscopy
GC–MS: Gas chromatography coupled to mass spectrometry
GPC/SEC: Gel permeation/size exclusion chromatography
L-Dopa: L-3,4-dihydroxyphenylalanine
OECD: Organisation for Economic Co-operation and Development
rMt: Recombinant laccase of *Myceliophtora thermophile*
PEG/PEO: Polyethylene glycol/polyethylene oxide
RP-HPLC-DAD: Reverse-phase HPLC coupled to a diode array detector
TMS: Trimethylsilyl
