# Supplementary material for: Production and properties of non-cytotoxic pyomelanin by laccase and comparison to bacterial and synthetic pigments
Source: Sci Rep. 2021 Apr 20;11:8538. doi: 10.1038/s41598-021-87328-2 (PMC8058095; doi:10.1038/s41598-021-87328-2)
Supplement: Supplementary file 1 — Supplementary Information [file 41598_2021_87328_MOESM1_ESM.docx]

Supplementary information

**Production and Properties of non-Cytotoxic Pyomelanin by Laccase and Comparison to Bacterial and Synthetic Pigments**

Faustine Lorquin^1,2^, Fabio Ziarelli^3^, Agnès Amouric^1^, Carole Di Giorgio^2^, Maxime Robin^2^, Philippe Piccerelle^2^, Jean Lorquin^1,*^

^1^ Aix-Marseille Université, Mediterranean Institute of Oceanology (MIO), 163 avenue de Luminy, 13288 Marseille cedex 9, France

^2^ Aix-Marseille Université, Mediterranean Institute of Marine and Terrestrial Biodiversity and Ecology (IMBE), 27 boulevard Jean Moulin, 13385 Marseille cedex 5, France

^3^ Aix-Marseille Université, Fédération Sciences Chimiques de Marseille, 52 avenue Escadrille Normandie Niemen, 13397 Marseille, France

**L-tyrosine degradation pathways**

**Fig. 1S**


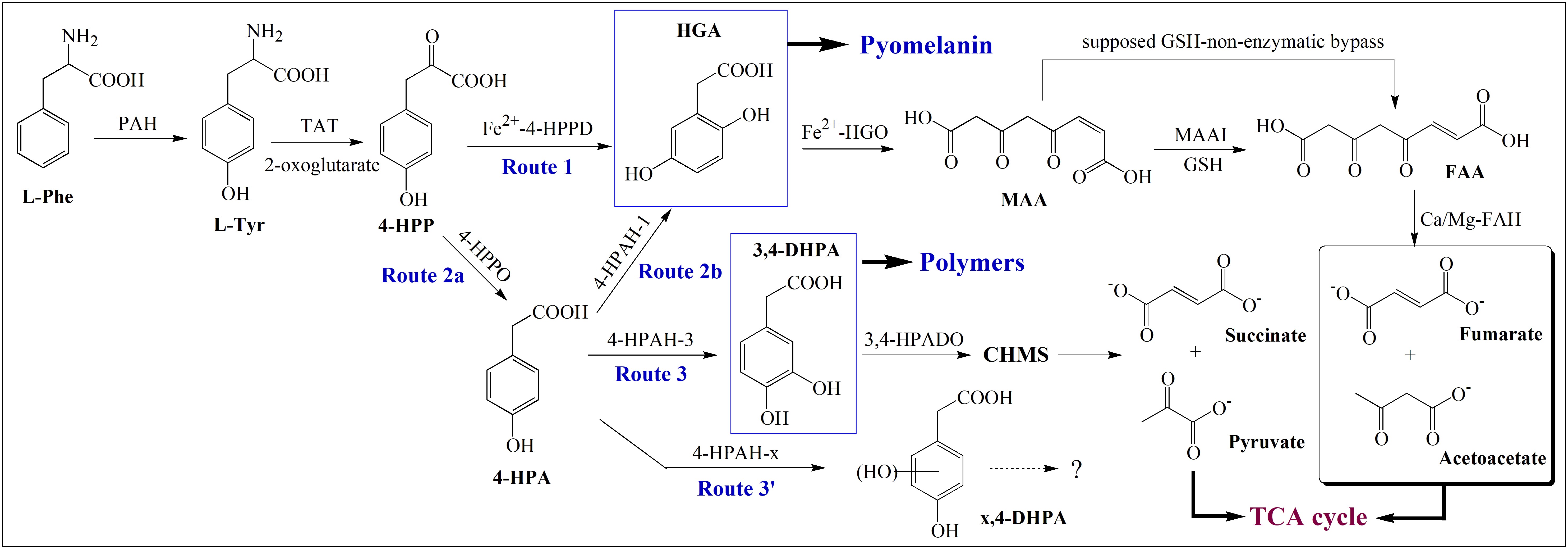


**Figure 1S**. L-tyrosine (L-Tyr) pathway in microorganisms and degradation of homogentisic acid (HGA). The pathway is firstly initiated by the common transamination of L-Tyr in 4-hydroxyphenylpyruvate (4-HPP) by an L-Tyr transaminase (TAT; EC 2.6.1.5) in presence of 2-oxoglutarate. From there, two distinct routes 1 and 2 led to HGA formation. In the most described route 1, 4-HPP is converted to HGA by a 4-hydroxyphenylpyruvate dioxygenase (4-HPPD, EC 1.13.11.27), a nonheme and Fe^2+^-dependant enzyme. In the less studied route 2 (a and b), 4-HPP is converted into 4-hydroxyphenylacetic acid (4-HPA) by a 4-hydroxyphenylpyruvate oxidase (HPPO, EC 1.2.3.13). From this, a 4-HPA-1-monooxygenase (4-HPAH-1, EC 1.14.13.18), a key NAD(P)H-dependent enzyme, hydroxylates the ring on C_1_ followed by a shift of the acetic group on C_2_ and leading to the HGA formation^22^. HGA is further converted into 4-maleylacetoacetic acid (MAA) by an HGA 1,2-dioxygenase (HGO, or HGA oxidase, EC 1.13.11.5) which opens the ring through an *ortho*-cleavage (C_1_-C_2_ cleavage). Then MAA is enzymatically isomerized in fumarylacetoacetate, this last finally transformed in fumarate and acetoacetate that breeds the Krebs cycle^34^. CHMS, 5-carboxymethyl-2-hydroxymuconic semialdehyde acid; DHPA, dihydroxyphenylacetic acid; FAA, fumarylacetoacetate; FAH, fumarylacetoacetate hydrolase (EC 3.7.1.2); 3,4-HPADO, 3,4-dihydroxyphenylacetate dioxygenase (EC 1.13.11.15); 4-HPAH-x, 4-dydroxyphenylacetic-x-hydroxylase (or monooxygenase, x=1-6); MAAI, maleylacetoacetate isomerase (EC 5.2.1.2); PAH, phenylalanine-4-hydroxylase (EC 1.14.16.1).

**Optimization of the enzymatic process (production of Pyo_ENZ_)**

**Summary.** To produce optimally Pyo_ENZ_, the conditions used are 40 mM HGA (final conc.), 17-22 U (~ 23-30 µL, syringaldazine assay) of rMt laccase extract per mL of reaction volume, at pH 6.8 (Na-phosphate buffer), for 24 h agitation (Fig. 2S and 3S) at 30°C. A temperature of 50°C does not improve the yield neither reduced the incubation time (Fig. 4S).

**Routine preparation of Pyo_ENZ_**. The process started from 1 g of 2,5-dimethoxyphenylacetic acid (CAS 1758-25-4). After demethylation by HBr (reflux) and evaporation, the resulting HGA-lactone is dissolved in 130 mL hot water (70°C), alkalinized at pH 9.3, and the solution immediately buffered at pH 6.8. Once the temperature of the solution reaches 30-40°C, the laccase is added, the solution agitated at 30°C for 48 h. The formed pigment is then precipitated by HCl (stayed 24 h), centrifuged, rinsed successively with milliQ-water (3x) and ethanol (1x), and dried.

**Fig. 2S**


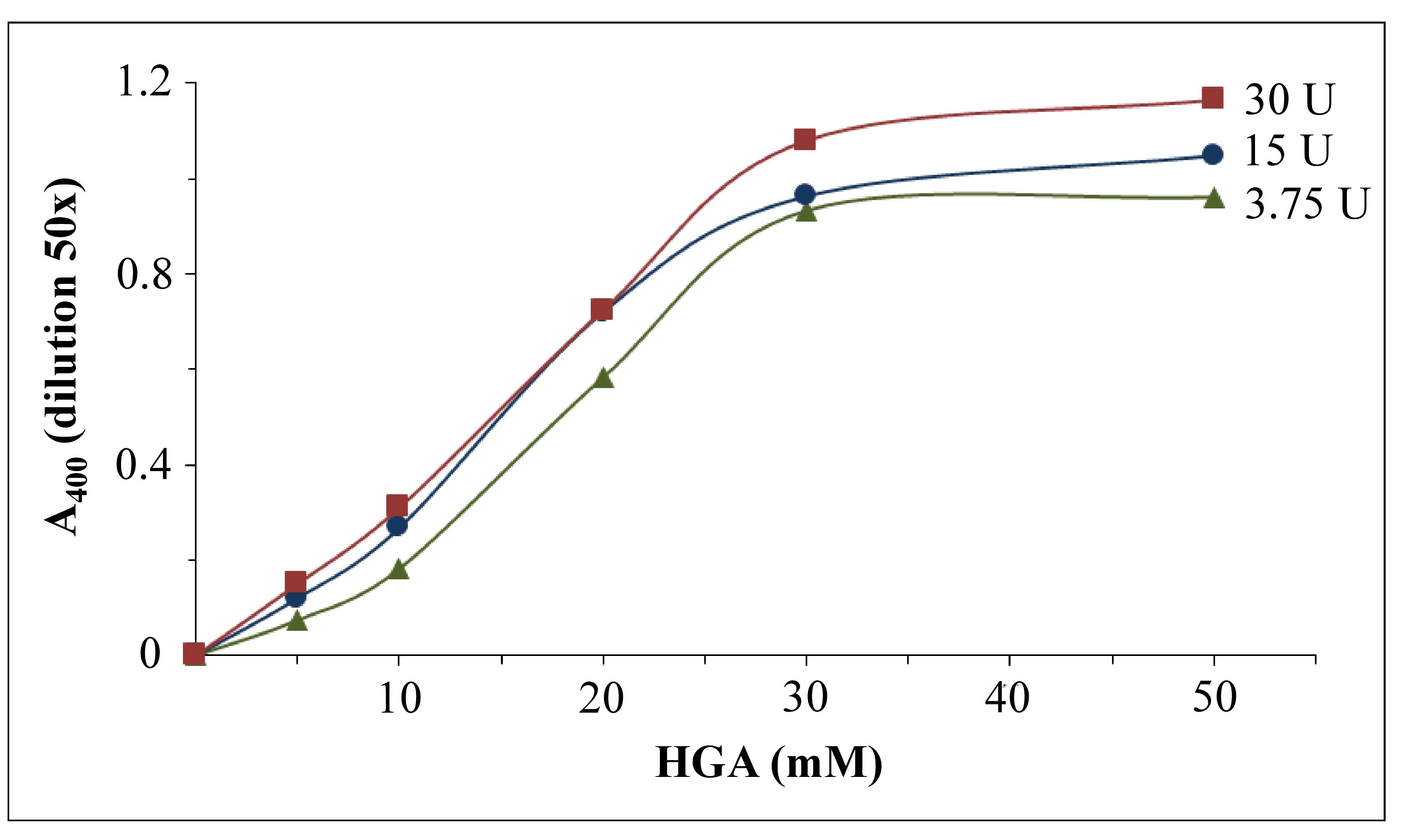


**Figure 2S**. Determination of the maximal HGA concentration for the pyomelanin synthesis by the rMt laccase at different activities. Conditions: reaction volume 1 mL at pH 6.8. SD ≤ 5% of the mean. The rMt laccase activity determination was adapted from the Sigma technical sheet. A 216 µM syringaldazine stock solution was prepared extemporaneously in MeOH and diluted 10x. The reaction was performed in a total volume of 1.5 mL and contained 1.1 mL of a 100 mM Na-phosphate buffer at pH 6.8 (73 mM final concentration), 250 µL of the laccase rMt, and the reaction started by the addition of 150 µL of the syringaldazine solution (2.16 µM final conc.). The absorbance at 530 nm (A_530_ sample) was read after 10 min incubation in dark at 30°C and compared to a blank performed in absence of laccase (A_530_ reference). Enzyme activity was expressed in Units/mL by using the formula:

(A_530_ sample - A_530_ reference)/0.001 x 0.25 x 10, where 0.001 corresponds to the A_530 nm_ variation per laccase unit at pH 6.8 at 30°C, 10 is the dilution factor of the substrate. The activity was determined in triplicate and standard deviations less than 5% of the mean.

**Fig. 3S**


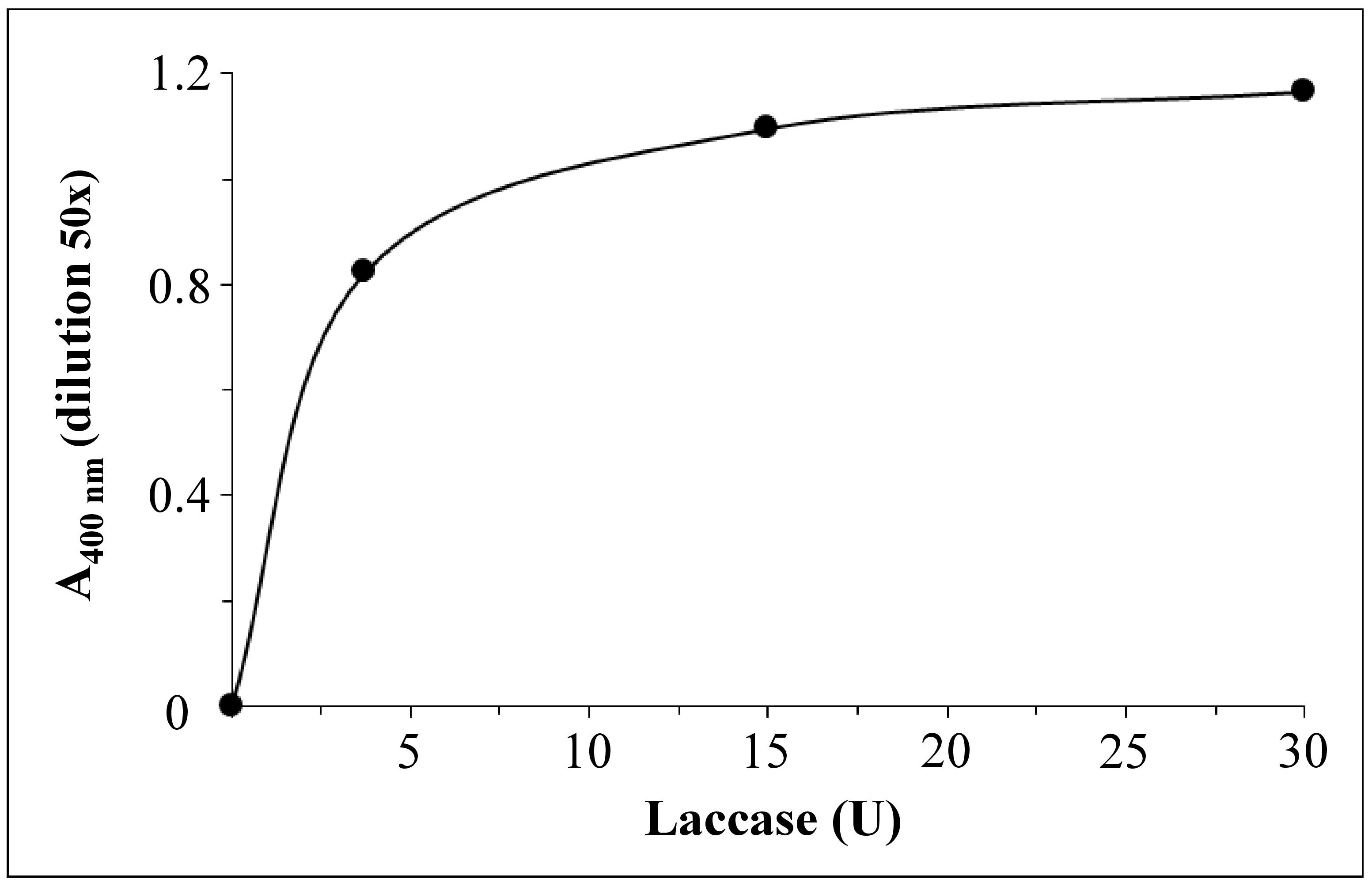


**Figure 3S**. Determination of the maximal rMt laccase activity for HGA polymerization. Conditions: reaction volume 1 mL, pH 6.8, HGA 40 mM, enzyme activity of the stock solution 750 U/mL (syringaldazine assay, see Fig. 2S). SD ≤ 5% of the mean.

**Fig. 4S**


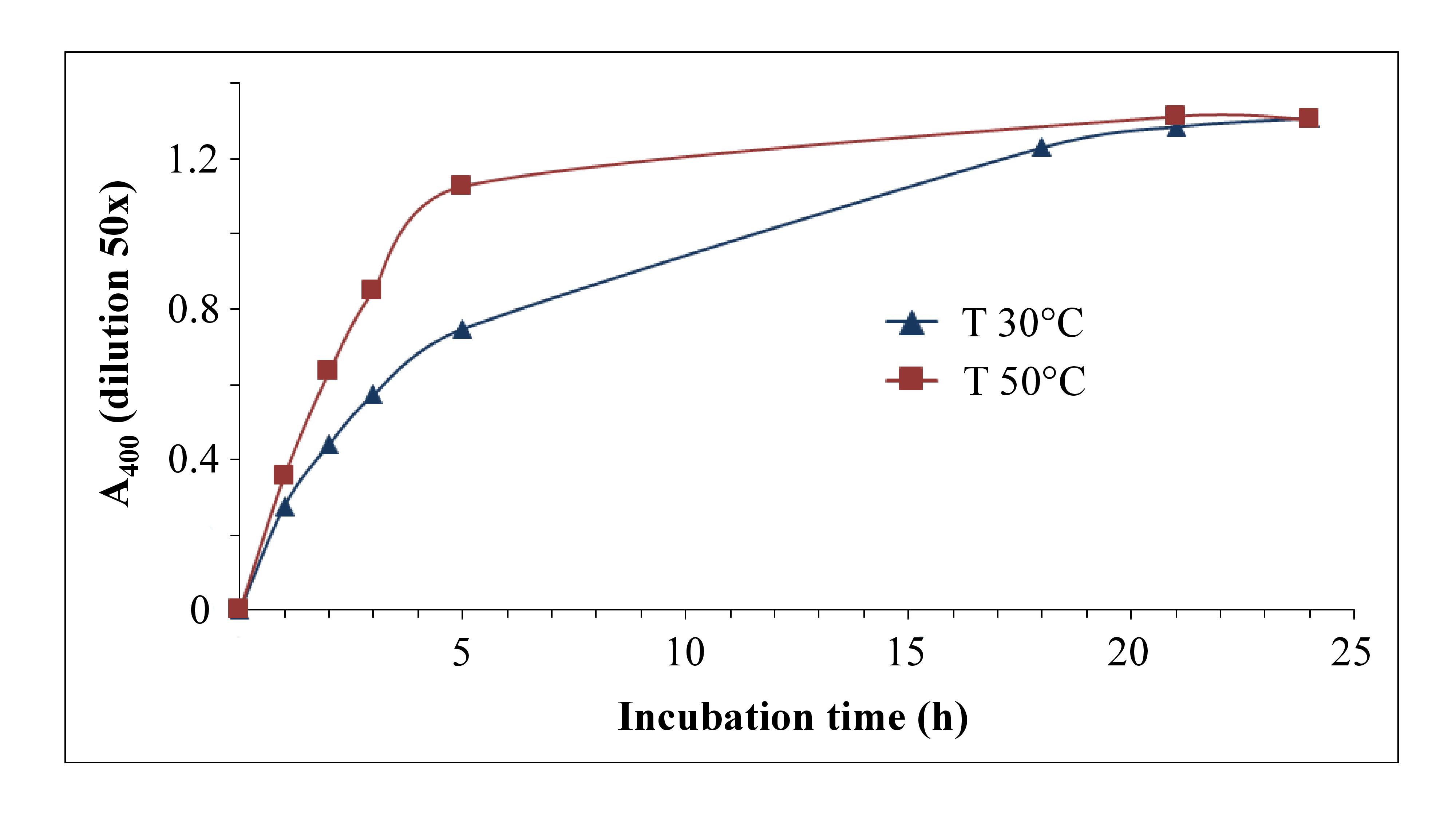


**Figure 4S**. Determination of the optimal incubation time and temperature on the polymerization of HGA. Conditions: reaction volume 1 mL, pH 6.8, HGA 40 mM, laccase activity 22 U per assay (syringaldazine assay, see Fig. 2S). SD ≤ 5% of the mean.

**Physicochemical properties of the pyomelanin**

**Fig. 5S**


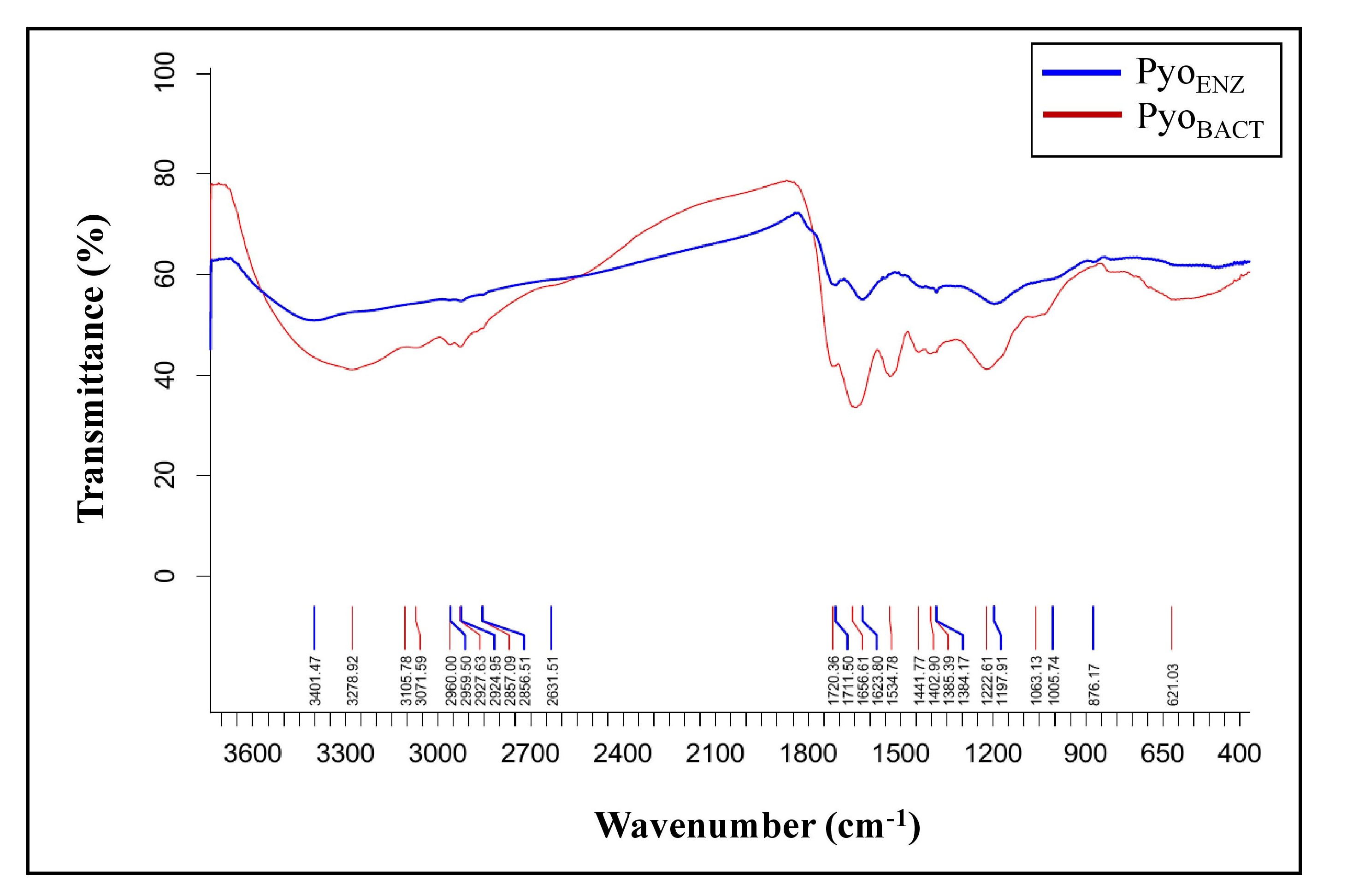


**Figure 5S**. Fourier-transform infrared (FTIR) spectra of pigments Pyo_ENZ_ (blue line) and Pyo_CHEM_ (red line). Spectra were recorded in the range of wavenumbers (cm^-1^) of 500-4,000 at a resolution of 4 cm^-1^, on a Vertex 70 FT-IR spectrometer (BRUKER OPTIK GmbH, Germany). Transmittance (T%) is expressed in terms of arbitrary units. Samples were prepared as KBr pellets. One

mg of pyomelanin was ground to homogeneity in a mortar together with 200

mg dry KBr and immediately pressed to a pellet (10

mm diameter) with 7.5 tons of pressure.

**Photostability of Pyo_ENZ_** (see protocol in Methods)

In NaOH at three diluted concentrations, the pigment has been exposed to drastic UVA-visible irradiation, 200 J/cm^2^ for one hour, a dose much higher than the minima imposed by the European Medicines Agency (72 J/cm^2^) for photostability studies of substances. It was concluded that Pyo_ENZ_ was highly photostable because no change was observed in the UV-visible spectrum (200-700 nm) of the polymer, comparatively to the non-irradiated sample, nor in its molecular weight assessed by SEC.

**Fig. 6S (A and B)**


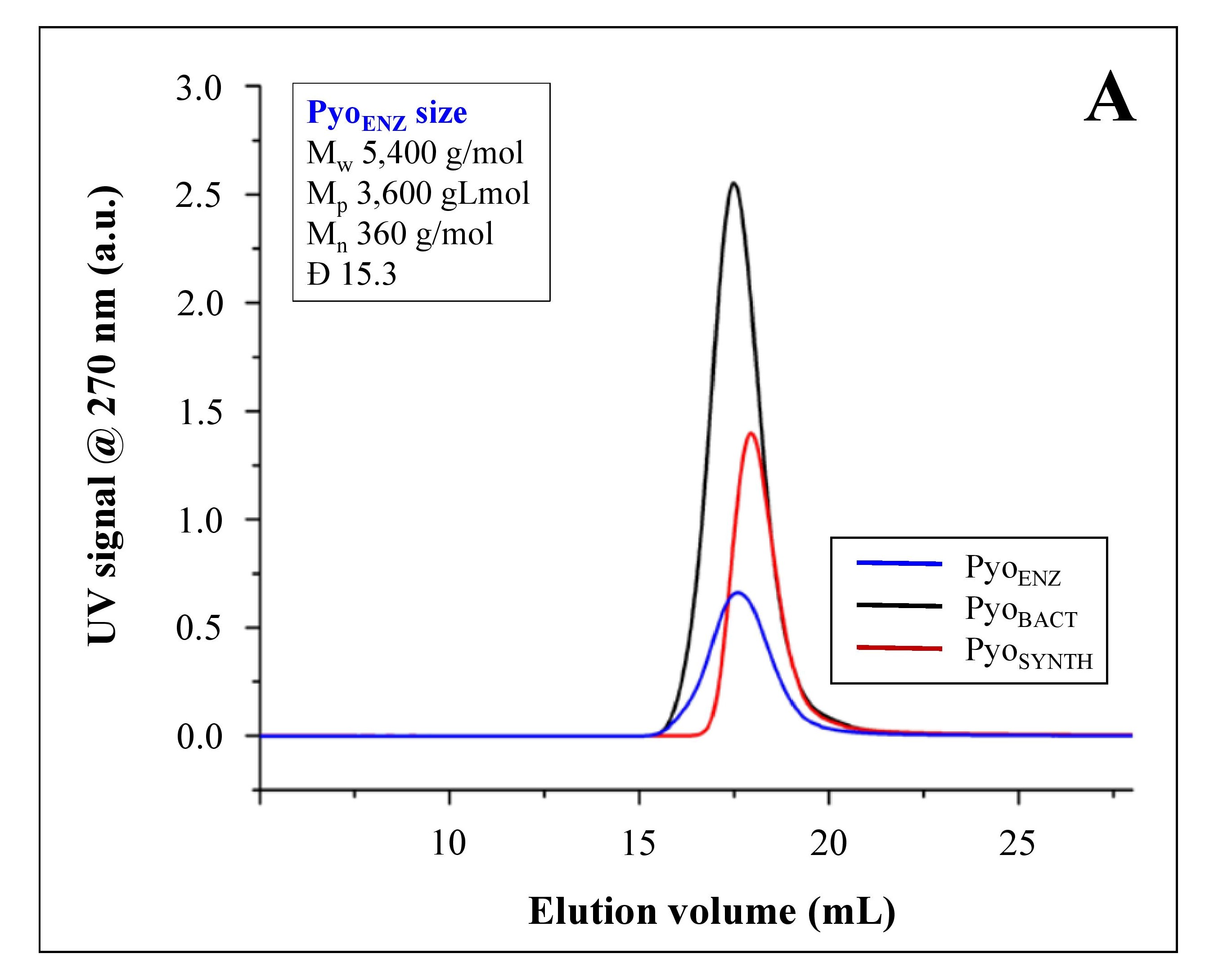


**Figure 6S**. (**A**) Size exclusion chromatography (SEC) elution profile of the three pyomelanin, Pyo_ENZ_, Pyo_BACT_, and Pyo_CHEM_; (**B**) Calibration curve built using Na-polystyrene sulfonate standards eluted in the same conditions as A. The determination was assessed on an HPLC WATERS system composed of a 515 pump, two detectors on-line, a 2487-UV set at 270 nm, a 2410-RI, and a 717plus autosampler, all controlled by an Empower GPC-upgraded software. The columns and the RI detector were maintained at 40°C. Separations were achieved on two MCX columns (300 x 8.0 mm, the first at 10^3^ and the second at 10^5^ Å pore size) from PSS (Perfect Separation Solution, Germany), and protected by a guard column (50 x 8 mm, 5 µm). About 2.5 mg of dried melanin was dissolved in 1 mL of NaOH 0.1 N containing 3 µL ethylene glycol (flow marker), from which 20 µL were injected for the isocratic separation using NaOH 0.1 N as eluent at a flow rate of 1 mL/min. Data were acquired by the software to display M_w_ (molecular weight), M_n_ (number average molecular weight), M_p_ (molecular weight of the highest peak), and Ɖ (dispersity, M_w_/M_n_) for each eluted polymer. The system was previously calibrated by Na-polystyrene sulfonate standards (from PSS, M_p_ 1,260 Da to 340 kDa) eluted in the same manner. A typical equation where v is the elution volume (in mL) is given in **B**. If the solubility of pyomelanin is not ensured, for instance with dimethylformamide (DMF)-LiCl 100 mM for sample preparation and eluent, the pigment forms high M_w_ agglomerates (~40-50 kDa) on aqueous or organic GPC/SEC supports and false the result.


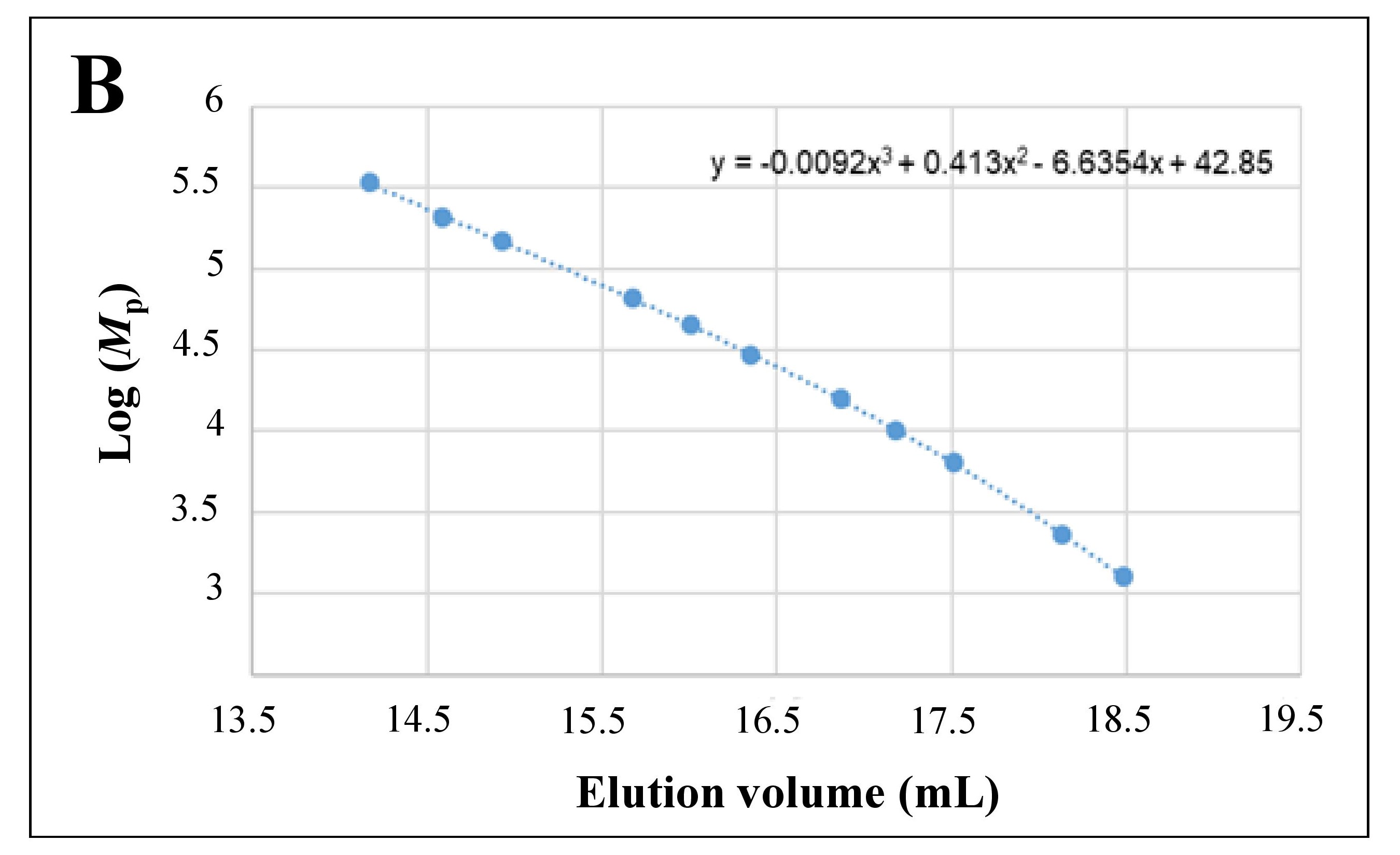


**DPPH-antioxidant activities of the pyomelanin comparatively to standards**

**Fig. 7S (A and B)**


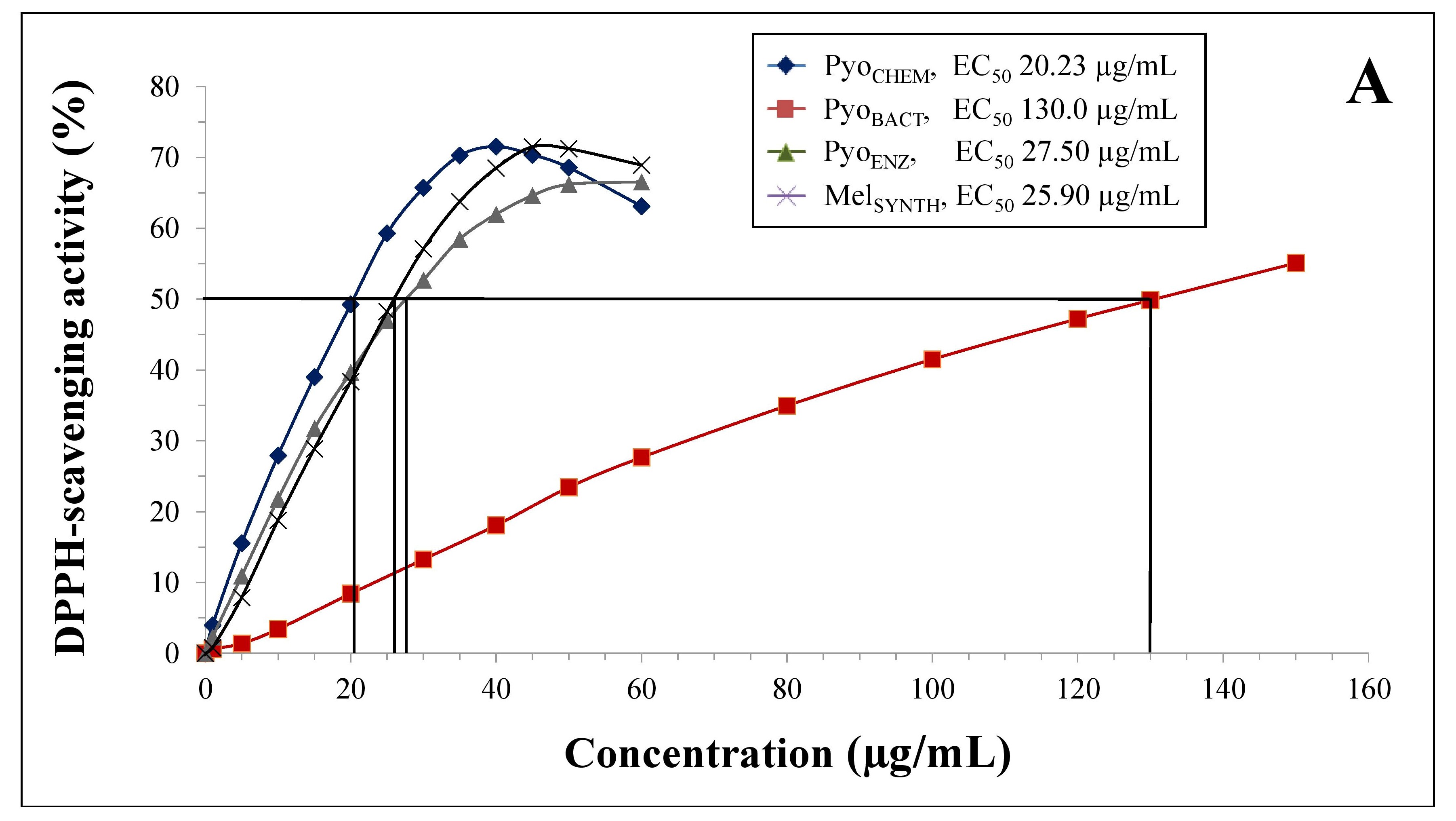

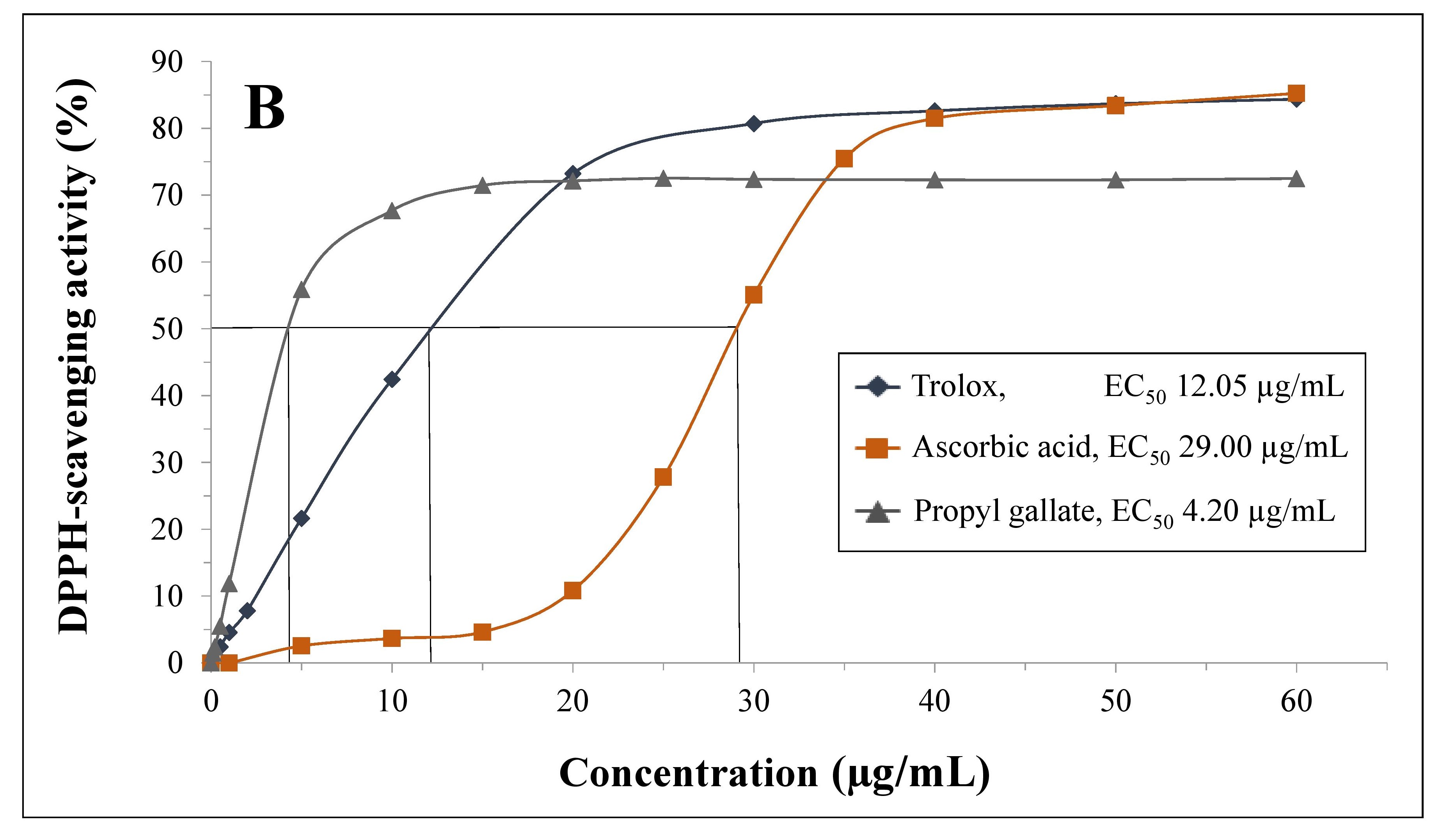


**Figure 7S.** DPPH free radicals scavenging effect of the purified pyomelanin expressed by the EC_50_ (in µg/mL), comparatively to the commercial melanin Mel_SYNTH_ (**A**), and standard antioxidants (**B**). All melanin was prepared at 0.5 mg/mL in DMSO by agitation for 24 h, at ambient temperature, with slight sonication (15 s, low power) at two-time intervals. Several concentrations of DPPH were tested to suitably determine the activity. The most convenient assay consisted of mixing 0.6 mL of DPPH 350 µM (in MeOH) with 0.3 mL of pigment in DMSO. The mixture was shaken vigorously, left to stand in the dark for 30 min at room temperature, and A_517 nm_ read against a blank of DPPH-DMSO (2:1 v/v). Antiradical standards such as Trolox (1 mg/mL stock solution), ascorbic acid (1 mg/mL), and propyl gallate (3,4,5-trihydroxybenzoic acid propyl ester; 0.25 mg/mL), all in DMSO, were used as positive controls and assayed at the range 0.5-60 µg each. The radical scavenging activity (RSA) was determined as the decrease in the A_517 nm_ and calculated using RSA (%) = [(A_control_ - A_sample_) / A_control_] x 100. RSA, and finally expressed as the EC_50_ value which represents the effective concentration of the test compound at which 50% of the DPPH radicals were scavenged. All assays were performed in triplicate, EC_50_ determined by the Prism 7.0 software.
